# DualGCN: a dual graph convolutional network model to predict cancer drug response

**DOI:** 10.1186/s12859-022-04664-4

**Published:** 2022-04-15

**Authors:** Tianxing Ma, Qiao Liu, Haochen Li, Mu Zhou, Rui Jiang, Xuegong Zhang

**Affiliations:** 1 MOE Key Laboratory of Bioinformatics, Bioinformatics Division, BNRIST and Department of Automation, Tsinghua University, Beijing, 100084 China; 2 Department of Statistics, Stanford University, Stanford, CA 94305 USA; 3 School of Medicine, Center for Synthetic and Systems Biology, Tsinghua University, Beijing, 100084 China; 4 SenseBrain Research, San Jose, CA 95131 USA

**Keywords:** Cancer drug response, Graph convolutional networks, Protein–protein interactions, Tumor heterogeneity

## Abstract

**Background:**

Drug resistance is a critical obstacle in cancer therapy. Discovering cancer drug response is important to improve anti-cancer drug treatment and guide anti-cancer drug design. Abundant genomic and drug response resources of cancer cell lines provide unprecedented opportunities for such study. However, cancer cell lines cannot fully reflect heterogeneous tumor microenvironments. Transferring knowledge studied from in vitro cell lines to single-cell and clinical data will be a promising direction to better understand drug resistance. Most current studies include single nucleotide variants (SNV) as features and focus on improving predictive ability of cancer drug response on cell lines. However, obtaining accurate SNVs from clinical tumor samples and single-cell data is not reliable. This makes it difficult to generalize such SNV-based models to clinical tumor data or single-cell level studies in the future.

**Results:**

We present a new method, DualGCN, a unified Dual Graph Convolutional Network model to predict cancer drug response. DualGCN encodes both chemical structures of drugs and omics data of biological samples using graph convolutional networks. Then the two embeddings are fed into a multilayer perceptron to predict drug response. DualGCN incorporates prior knowledge on cancer-related genes and protein–protein interactions, and outperforms most state-of-the-art methods while avoiding using large-scale SNV data.

**Conclusions:**

The proposed method outperforms most state-of-the-art methods in predicting cancer drug response without the use of large-scale SNV data. These favorable results indicate its potential to be extended to clinical and single-cell tumor samples and advancements in precision medicine.

**Supplementary Information:**

The online version contains supplementary material available at 10.1186/s12859-022-04664-4.

## Background

Anti-cancer drugs have played important roles in cancer therapy in recent years. However, the occurrence of drug resistance limits the effectiveness of anti-cancer drugs [1]. It is essential to fully explore the cancer drug response (CDR) underlying comprehensive biological systems.

Cancer drug response can be studied with cancer cell line models. Drug response on these models is quantitatively described by the half-maximal inhibitory concentration (IC50). The IC50 depicts the amount of drug needed to inhibit cancer cell growth by half. A smaller IC50 indicates that the drug is relatively more powerful. Comprehensive genetic and pharmacologic characterizations of cancer cell line models are collected by projects such as Cancer Cell Line Encyclopedia (CCLE) [2], Catalogue of Somatic Mutations in Cancer (COSMIC) [3], and Genomics of Drug Sensitivity in Cancer (GDSC) [4]. Such data enable researchers to develop predictive machine learning models of anti-cancer drug sensitivity [5–9]. These models consist of two parts that are responsible for encoding drugs and cell lines separately. Drugs are represented through one-hot encoding using simplified molecular-input line-entry system (SMILES) data [7, 8]. Genomic mutations have been reported to have significantly different patterns across cell lines [4]. They are widely used as features of cancer cell lines, and are encoded by models such as multilayer perceptrons (MLP) [7] and convolutional neural networks (CNN) [8, 9]. However, drug resistance could not be fully discovered using these in vitro cancer cell lines. It has been revealed that tumors are highly heterogeneous [10], and tumor microenvironments have essential influences on tumor progression [11–13]. Such heterogeneity and interaction could not be reflected with in vitro cancer cell lines only. Emerging single-cell data and clinical data show the potential to decipher complex tumor microenvironments and to unlock drug response [14–16]. Transferring knowledge studied from in vitro cancer cell lines to single-cell and clinical data is a promising avenue [14].

There are some limitations in current methods to be generalized to single-cell and clinical data. First, most existing methods include SNVs as features to improve the predictive ability on cancer cell lines. However, it has been revealed that calling SNVs reliably from cancer samples cannot always be reached. High-frequency genomic aberrations and aneuploidy are common in cancers, and these variations reduce SNV detection efficiency [17]. Similarly, detecting reliable SNVs covering all hotspots simultaneously from single-cell data is unattainable. Both sequencing coverage and sequencing depth in single-cell data are too low to detect SNVs completely from the data [18, 19]. Second, current methods encode gene features as separate units. However, recent evidence from single-cell studies shows that the tumor microenvironment is a complex system [11]. Tumor cells interact with surrounding cells. Such interactions form a biological network, and the whole ecosystems contribute to drug response simultaneously [20–22]. These inspired us to develop new methods without using SNVs as features and considering cancer samples as systems with interactions between proteins.

In this paper, we propose a novel deep learning model called DualGCN. It consists of dual graph convolutional networks (GCN) [23] and takes drug structures and omics data as input to predict cancer drug response. One GCN module learns intrinsic chemical features of drugs. Nodes in this module represent atoms of drugs, and edges indicate connections between the atoms. Meanwhile, the other GCN module incorporates protein–protein interactions (PPI) and extracts the underlying biological features of cancer samples. Nodes in this module represent proteins, and edges indicate protein–protein interactions. In this study, we used gene expression and copy number variation as gene features. These features have been demonstrated to be vital to depict cancer cell types in recent single-cell studies [24–28]. We conducted extensive experiments and demonstrated that our method outperforms most state-of-the-art methods while avoiding the use of SNVs. In addition, we conducted a case study on clinical cancer patients with DualGCN and showed its potential to be extended to clinical and single-cell cancer samples.

## Results and discussion

### Overview of DualGCN

**Fig. 1:**
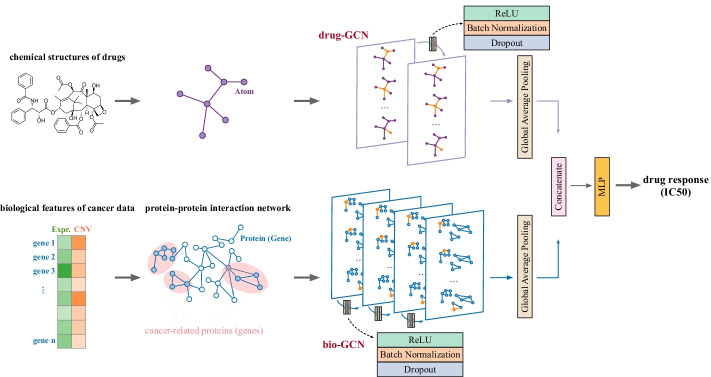
Overview of DualGCN. DualGCN takes chemical structure information of a drug and gene features of a cancer sample as inputs to the (1) drug-GCN module and (2) bio-GCN module, respectively. It outputs the response (IC50) of the given drug on the given cancer sample. (1) In the drug-GCN module, drug chemical structure data are first transformed using the previous algorithm [29]. The transformed features are considered as features of nodes (atoms). Edges between nodes represent connections between atoms of drugs. (2) The bio-GCN module is built based on PPI networks where nodes indicate cancer-related proteins (genes) and edges represent interactions between proteins. This module takes gene expression and copy number variation of cancer-related genes as inputs. Such gene features are considered as features of corresponding nodes. Embeddings from the two GCN modules are then concatenated and fed into MLP to study cancer drug response

DualGCN takes chemical structure data of drugs and gene features of cancer samples as inputs and outputs drug response (IC50). The concept of DualGCN is shown in Fig. 1. The top panel of Fig. 1 is a GCN module (named drug-GCN below) used to encode the drug chemical structure. Nodes in this module represent atoms of drugs. Edges between nodes indicate connections between the atoms of drugs. Features of atoms are learned from the previous algorithm [29]. The bottom panel of Fig. 1 is another GCN module (named bio-GCN below) used to encode biological features of cancer samples. It is built on PPI networks and takes features of cancer-related genes as inputs. We used gene expression (Expr.) and copy number variation (CNV) as gene features in this study. These gene features were demonstrated to have important roles in decoding cancer cell types from recent studies [26–28]. Both GCN modules use ReLU as activation functions and adopt batch normalization [30] and dropout [31] strategies to improve model robustness. Two embeddings from the drug-GCN module and the bio-GCN module are then concatenated together to be fed into a multilayer perceptron to study the response of the given drug on the given cancer sample. Detailed settings of the model can be found in Additional file 1: Table S1.

### Assessment of methods

We evaluated the performance of DualGCN as well as baselines including support vector machine (SVM), random forest, Lasso regression, ridge regression, CDRscan [7], and DeepCDR [8]. The evaluation was conducted on 86,530 drug-cell line pairs. These data included 208 drugs and 525 cell lines covering 27 kinds of cancers. Data preparation and configurations of baselines are described in the “Methods” section. The evaluation was conducted with five-fold cross-validation (CV). We used evaluation metrics including Pearson’s correlation coefficient, Spearman’s correlation coefficient, and root mean square error (RMSE).

DualGCN achieves strong predictive performance without the use of SNVs. It gained Pearson’s correlation = 0.925, Spearman’s correlation = 0.907, and RMSE = 1.079. It significantly outperformed traditional methods, including SVM, random forest, Lasso regression, and ridge regression (Table 1). Detailed configurations and results of these methods can be found in Additional file 1: Table S5, Additional file 1: Table S6, and Additional file 1: Table S7. In addition, we also compared DualGCN with deep learning models. DualGCN had consistent improvements over CDRscan among all evaluation metrics. Improvements in Pearson’s correlation, Spearman’s correlation, and RMSE were 0.014, 0.013, and 0.094, respectively. DeepCDR gained higher predictive performance than DualGCN. The differences in Pearson’s correlation, Spearman’s correlation, and RMSE were 0.003, 0.003, and 0.013, respectively. Such differences needed huge SNV information. DeepCDR contains several sub-networks encoding multi-omics data. We evaluated its performance without SNV by removing the corresponding sub-network and denoted it by DeepCDR (-). Pearson’s correlation, Spearman’s correlation, and RMSE of DeepCDR (-) dropped to 0.900, 0.877, and 1.265, respectively. DualGCN gained a large margin over it without tens of thousands of SNVs. Improvements in Pearson’s correlation, Spearman’s correlation, and RMSE are 0.025, 0.030, and 0.186, respectively. There are two major reasons SNV data should be treated with caution. First, different projects collected SNVs in different patterns and used different references (human reference genome or normal tissues) in SNV calling algorithms. Thus, SNVs might not be aligned across data from different sources. Second, studying drug responses on in vitro cancer cell lines only cannot fully reveal the mechanisms of drug resistance. Transferring knowledge studied from in vitro cancer cell lines to single-cell and clinical data tends to be an important direction [14]. However, it is unreliable to call SNVs from clinical and single-cell tumor data covering all candidate loci [17–19]. In addition, recent evidence shows that whole tumors collectively act on drugs [12]. These studies gradually accumulate protein–protein interactions influencing cancer progression and drug response [13]. DeepCDR encodes different features of the same unit (gene) separately. It is difficult for such encoding systems to further include constantly discovered and important interacting protein pairs. DualGCN encodes genes as basic units. It achieves strong predictive performance without SNV data. Such advances indicate its potential to absorb new biological knowledge and to be generalized to studies on clinical data and at single-cell resolution.

**Table 1:** Performance comparison

Method	Pearson’s correlation	Spearman’s correlation	RMSE
SVM	0.336 ± 0.078	0.230 ± 0.071	3.115 ± 0.053
Random Forest	0.864 ± 0.001	0.839 ± 0.003	1.441 ± 0.008
Lasso	0.893 ± 0.002	0.873 ± 0.002	1.284 ± 0.007
Ridge	0.895 ± 0.002	0.875 ± 0.002	1.268 ± 0.007
DeepCDR (-)	0.900 ± 0.004	0.877 ± 0.004	1.265 ± 0.020
CDRscan	0.911 ± 0.002	0.894 ± 0.002	1.173 ± 0.011
DualGCN	0.925 ± 0.001	0.907 ± 0.002	1.079 ± 0.007
DeepCDR	0.928 ± 0.001	0.910 ± 0.001	1.066 ± 0.004

**Fig. 2:**
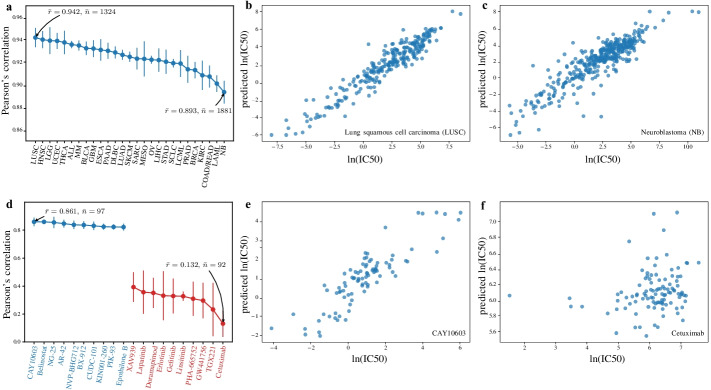
Performance of DualGCN across cancers and drugs. **a** Pearson’s correlation on each type of cancer. We calculated the average Pearson’s correlation coefficients of samples belonging to each type of cancer and sorted the coefficients from large to small (from left to right in the figure). Blue dots indicate the mean of Pearson’s correlation across CVs and are denoted by $\overline{r}$. Vertical blue bars represent variances of Pearson’s correlation across CVs. $\overline{n}$ denotes average sample size across CVs. The largest and smallest Pearson’s correlation coefficients were obtained on lung squamous cell carcinoma (LUSC) and neuroblastoma (NB), respectively. **b** Scatterplot of correlations between true and predicted IC50 on LUSC. **c** Scatterplot of correlations between true and predicted IC50 on NB. **d** Pearson’s correlation on each drug. We calculated the average Pearson’s correlation coefficients of samples belonging to each drug and sorted the coefficients from large to small. The left ten in the figure are drugs with the best predictive performance, and the right ten are drugs with the worst predictive performance. Blue dots indicate the mean of Pearson’s correlation across CVs and are denoted by $\overline{r}$. Vertical blue bars represent variances of Pearson’s correlation across CVs. $\overline{n}$ denotes average sample size across CVs. The largest and smallest Pearson’s correlation coefficients were obtained on CAY10603 and cetuximab, respectively. **e** Scatterplot of correlations between true and predicted IC50 on CAY10603. **f** Scatterplot of correlations between true and predicted IC50 on cetuximab

DualGCN achieves high performance across different types of cancers consistently. Pearson’s correlation coefficients on different cancers ranged from 0.942 to 0.893 (Fig. 2a). The highest and the lowest coefficients were obtained on lung squamous cell carcinoma (LUSC) and neuroblastoma (NB), respectively. Scatterplots of these two cases are shown in Fig. 2b and Fig. 2c. We also evaluated the performance across drugs. Pearson’s correlation coefficients for different drugs varied in a wide range from 0.861 to 0.132 (Fig. 2d). The highest and the lowest coefficients were obtained on CAY10603 and cetuximab, respectively. Scatterplots of these two cases are shown in Fig. 2e, f. We performed principal component analysis (PCA) on SMILEs of drugs. We observed that latent representations of CAY10603 and cetuximab were close in low-dimensional space. This result indicates that the structures of these two drugs are similar, though the prediction performances on these two drugs were significantly different (Additional file 1: Figure S2). In addition, we found that the IC50 of cetuximab was much higher than that of other drugs. These findings indicate that drugs with low prediction performances may be affected by their isolation of IC50 from the overall distribution.

### Ablation analysis

We conducted ablation studies to evaluate the effects of different gene features on DualGCN. We studied such effects by taking only one kind of features as the input. The results are shown in Table 2. CNV data contributed more than gene expression data to our model. In addition, simultaneously taking gene expression and CNV data gained higher predictive performance than single kind of features.

**Table 2:** Ablation study on gene features

	Pearson’s correlation	Spearman’s correlation	RMSE
Expr.	0.908 ± 0.005	0.887 ± 0.008	1.191 ± 0.031
CNV	0.911 ± 0.007	0.892 ± 0.007	1.172 ± 0.046
Expr. + CNV	0.925 ± 0.001	0.907 ± 0.002	1.079 ± 0.007

### A case study on clinical cancer patients

We conducted a case study on clinical BRCA patients using the trained DualGCN model. Gene features and drug response annotations of patients were obtained from The Cancer Genome Atlas Program (TCGA) [32]. There is a noticeable difference in analyzing drug response from in vitro cancer cell lines and clinical cancer data. Drug response annotations of clinical cancer data are qualitatively described as grades. In contrast, responses on cancer cell lines are quantitatively depicted by the IC50. We first binarized the clinical drug response annotations of patients into “sensitive” and “resistant”. Such binary labels were considered as ground truth. Then, we predicted the drug responses of patients and calculated the corresponding drug sensitivity score (DSS). A high DSS indicates sensitivity, and a low DSS indicates resistance. Detailed descriptions of annotation transformation and definitions of the DSS are given in the “Methods” section. We set DSS on cancer samples as discrimination thresholds of the receiver operating characteristic (ROC) curve. We observed a modest consistency between the predicted drug responses and clinical annotations. The area of the curve (AUC) of the ROC curve was 0.661 (95% confidence interval: 0.558 to 0.765, shown in Additional file 1: Figure S3. Future studies may need to combine single-cell cancer data and cellular interactions to further decode cell-type composition and cancer drug resistance mechanisms.

## Conclusions

Anti-cancer drugs have played important roles in cancer treatments. However, resistance to anti-cancer drugs continues to be a serious challenge. Studying drug response on tumors is essential to improve the treatment of cancers and guide anti-cancer drug design. Cancer cell line models have been widely used for such research. However, tumors are heterogeneous and consist of different cell types and complex interactions. Studying in vitro cancer cell lines only cannot fully decode the mechanisms of drug resistance. Emerging single-cell technologies are powerful toolkits to explore cell-type composition and cellular interactions in tumors. Transferring drug response knowledge obtained from cell line models to clinical and single-cell data is an important direction. Single nucleotide variants are widely used as features of cancer cell lines in current cancer drug response studies. However, detecting SNVs covering all candidate genomic loci from clinical tumor data is not always reliable, let alone from single-cell data. Such SNV-based models are hard to extend to studies on clinical data and at single-cell resolution.

In this study, we developed a unified dual graph convolutional network model, DualGCN, to predict cancer drug response. DualGCN encodes both drugs and cancer samples using graph convolutional networks with protein–protein interactions embedded. We demonstrated that DualGCN gained high predictive abilities without the use of SNV data. Such advances indicate its potential to be further extended to clinical and single-cell data. Meanwhile, recent single-cell tumor studies have constantly discovered important interactions in tumors. DualGCN sets genes as units of the encoding system with links across them. Such structures make it easy to absorb newly discovered protein interactions essential to tumor progression and drug resistance. We organized a case study on analyzing clinical cancer samples using knowledge learned from cell line models, and observed a modest consistency between the predicted drug responses and clinical annotations.

In addition, we notice limitations of the proposed method. Units of the module encoding cancer samples are genes. Thus, input features are at the gene level. Such structures provide a convenient interface to combine interacting protein pairs constantly discovered from cancer research. However, other non-gene level signals, such as histone modifications, are hard to encode into the module directly.

In summary, we introduce a method, DualGCN, that achieves high predictive abilities on cancer drug response without using SNV data. The method could be extended to clinical and single-cell data and has the potential to promote the development of precision medicine.

## Methods

### Drug and cell line data preparation

Drug data were downloaded from the GDSC (version: GDSC1) [4]. We only kept drugs that were recorded in PubChem [33]. In addition, drugs sharing the same PubChem identifiers but owning different GDSC identifiers were also filtered out. Finally, we collected 208 drugs. Detailed descriptions of these drugs can be found in Additional file 1: Table S2. We then transformed drug chemical structure data to obtain feature vectors of atoms of drugs using the previous algorithm [29]. Dimension of these feature vectors was $l_{d} = 75$. It has been proved that these feature vectors reflect the intrinsic properties of drugs, such as atom type, atom connectivity, and degrees of freedom.

Gene features of cancer cell lines were downloaded from CCLE (version: 19Q2) [2]. We filtered out cell lines if (1) either gene expression or CNV data were unavailable, or (2) cancer type annotations were missed, or (3) the sample size of the corresponding cancer type was less than 10. Finally, we collected 525 cell lines covering 27 kinds of cancers. Detailed descriptions of these cell lines can be found in Additional file 1: Table S3. Gene expression data were represented as $log_{2} \left( {TPM + 1} \right)$. CNV data were represented as $log_{2} \left( {CN + 1} \right)$, where $CN$ represents the relative copy number. We then used z-score normalization on these gene features.

Cancer drug response data (IC50) were downloaded from GDSC (version: GDSC1) [4]. The IC50 describes the amount of drug needed to inhibit cancer cell growth by half. In GDSC, the IC50 is recorded in the scale of µM and is transformed with natural logarithm. Finally, we collected 86,530 drug-cell line pairs.

### Construction of drug-GCN module

Drug-GCN module takes feature and adjacency matrix of drugs as inputs. It considers each drug as a graph where nodes represent atoms of the drug and edges indicate connections between atoms. This module extracts intrinsic chemical attributes using the graph convolutional network algorithm [23]. Different drugs have different number of atoms (from 5 to 96 in this study), so the scales of these raw drug graphs $G_{{d \text{-}raw}}$ vary. We first built a fixed-scale graph $G_{d}$, and then embedded the raw drug graph $G_{{d \text{-}raw}}$ into it. Such operations ensure that the drug-GCN module is unified to all drugs. The number of nodes $N_{d}$ of graph $G_{d}$ is 100.

Mathematically, raw drug graph $G_{{d \text{-}raw\left( i \right)}} = \left( {X_{{d \text{-}raw\left( i \right)}} , A_{{d \text{-}raw\left( i \right)}} } \right)$ is a sub-graph of the fixed-scale graph $G_{d\left( i \right)} = \left( {X_{d\left( i \right)} , A_{d\left( i \right)} } \right)$. Additional nodes in $G_{d\left( i \right)}$ are filled with zeros,

$$X_{d\left( i \right)} = \left( {\begin{array}{*{20}l} {X_{{d \text{-}raw\left( i \right)}} } \hfill \\ {0_{c1\left( i \right)} } \hfill \\ \end{array} } \right)\quad A_{d\left( i \right)} = \left( {\begin{array}{*{20}l} {A_{{d \text{-}raw\left( i \right)}} } \hfill & {0_{c2\left( i \right)} } \hfill \\ {0_{c3\left( i \right)} } \hfill & {0_{c4\left( i \right)} } \hfill \\ \end{array} } \right)$$

where $X_{d\left( i \right)} \in {\mathbb{R}}^{{N_{d} \times l_{d} }}$ denotes the feature matrix of the fixed-scale graph $G_{d\left( i \right)}$. $A_{d\left( i \right)} \in {\mathbb{R}}^{{N_{d} \times N_{d} }}$ denotes binary adjacency matrix of $G_{d\left( i \right)}$. Similarly, $X_{{d \text{-}raw\left( i \right)}} \in {\mathbb{R}}^{{N_{i} \times l_{d} }}$ and $A_{{d \text{-}raw\left( i \right)}} \in {\mathbb{R}}^{{N_{i} \times N_{i} }}$ denote the feature matrix and adjacency matrix of $G_{{d \text{-}raw\left( i \right)}}$, respectively. $N_{i}$ denotes the number of atoms of drug $i$. $0_{c1\left( i \right)}$, $0_{c2\left( i \right)}$, $0_{c3\left( i \right)}$, and $0_{c4\left( i \right)}$ are zero matrices.

According to the GCN algorithm [23], we have,

1 $$H_{d}^{{ \left( {l + 1} \right)}} = {\text{ReLU}}\left( {\tilde{D}_{d}^{{ - \frac{1}{2}}} \tilde{A}_{d} \tilde{D}_{d}^{{ - \frac{1}{2}}} H_{d}^{\left( l \right)} W_{d}^{\left( l \right)} } \right)$$

where $H_{d}^{\left( l \right)}$ is the output of layer $l$, and $H_{d}^{\left( 0 \right)}$ is the initial feature matrix $X_{d}$. $\tilde{A}_{d} = A_{d} + I_{d}$ is a modified adjacency matrix with self-connections. $I_{d}$ is an identity matrix. Diagonal matrix $\tilde{D}_{d}$ is a degree matrix of $\tilde{A}_{d}$ with $\tilde{D}_{d} \left[ {k,k} \right] = \mathop \sum \limits_{m} \tilde{A}_{d} \left[ {k,m} \right]$. $W_{d}^{\left( l \right)}$ represents weights of the layer $l$.

Detailed configurations of the drug-GCN module can be found in Additional file 1: Table S1.

### Construction of bio-GCN module

Bio-GCN module takes the gene features of cancer samples as inputs. Gene expression and CNV data were used in this study. These gene features were first fed into a two-layer MLP and the latent features were considered as features of genes. The module considers each cancer sample as a graph where nodes are proteins (genes) and edges indicate interactions between proteins. Such protein–protein interaction information was obtained from the STRING database (version 11.0, Taxonomy ID: 9606) [34]. Meanwhile, we only kept proteins that are known to be related to cancers. Such cancer-related proteins (genes) were collected from COSMIC [3] and TCGA [32]. We finally obtained 697 cancer-related genes (Table S4 in Additional file 1) and 55,140 protein–protein interaction pairs among them.

Mathematically, the biological graph of cancer sample $j$ is denoted by $G_{b\left( j \right)} = \left( {X_{b\left( j \right)} , A_{b\left( j \right)} } \right)$. $X_{b\left( j \right)} \in {\mathbb{R}}^{{N_{b} \times l_{b} }}$ and $A_{b\left( j \right)} \in {\mathbb{R}}^{{N_{b} \times N_{b} }}$ denote the feature matrix and adjacency matrix, respectively. $N_{b}$ denotes the number of nodes. $l_{b}$ denotes dimension of features of genes. $A_{b\left( j \right)}$ is a symmetric binary matrix. $A_{b\left( j \right)} \left[ {k,m} \right] = A_{b\left( j \right)} \left[ {m,k} \right] = 1$ if gene $k$ and gene $m$ have interactions in the PPI network. Otherwise, $A_{b\left( j \right)} \left[ {k,m} \right] = A_{b\left( j \right)} \left[ {m,k} \right] = 0$.

Then, the bio-GCN module uses graph convolutional network algorithms to extract intrinsic biological features of the cancer sample. The formula is as same as Eq. (1). Detailed configurations of the bio-GCN module can be found in Additional file 1: Table S1.

### Configurations of baselines

We compared DualGCN with six baselines, including DeepCDR [8], CDRscan [7], SVM, random forest, Lasso regression, and ridge regression. We additionally collected SNV data from the CCLE because they were necessary when using some of the baselines. We finally collected 27,180 SNVs within the cancer-related genes. We encoded the SNV features as binary vectors with one denoting the occurrence of a mutation.

DeepCDR [8] encodes multi-omics data using CNN separately. Genomic features including SNVs, gene expression, and copy number variation were used. Besides, it encodes drug data using graph convolutional networks. Meanwhile, we also tested the performance of DeepCDR without using SNV data by removing the corresponding CNN module. This modified version is denoted by DeepCDR (-). CDRscan [7] encodes SNVs using CNN. Besides, drugs are represented through one-hot encoding on SMILES data. SMILES is a string where characters represent atoms and connectivity relationships. We obtained SMILES (isomeric type) of drugs through parsing related XML files from PubChem. In addition, we also tested SVM, random forest, Lasso regression, and ridge regression using SNVs as features of cell lines, and drugs were represented through one-hot encoding of SMILES. We applied kernels including radial basis function (RBF) kernel, polynomial kernel, and sigmoid kernel for SVM. We applied multiple number of trees (n = 50, 100, 200) for random forest. We set coefficient alpha = 0.01, 0.1, 0.5 for Lasso regression. We set coefficient alpha = 0.1, 0.5, 1.0, 2.0 for ridge regression.

### Clinical cancer data preparation

We conducted a case study on clinical cancer patients using DualGCN. First, we curated data of patients whose drug response information was available in TCGA. Patients with breast invasive carcinoma (BRCA) owned the largest scale (195 records) and were included in this case study. Then, we downloaded the gene features of these cancer patients through Firehose Broad GDAC (http://gdac.broadinstitute.org/). Gene expression data of patients were transformed as $log_{2} \left( {TPM + 1} \right)$. CNV data were at segment-level originally. We further transformed these segment-level CNV data into gene-level. There are $K$ segments overlapping some gene, and the length of each overlapped region is denoted by $l_{s} { }\left( {s = 1, 2, \ldots ,K} \right)$. Length of the gene is denoted by $L$. The relative copy number ratio of each segment is denoted by $c_{s} \left( {s = 1, 2, \ldots ,K} \right)$. We extracted the locations of genes from Ensembl (GRCh37) [35]. We transformed segment-level CNV data into gene-level and adopted logarithmic transformation using the following formula,

$$log_{2} \left( {\mathop \sum \limits_{{s = \left\{ {1, 2, \ldots ,K} \right\}}} c_{s} \frac{{l_{s} }}{L} + \left( {1 - \mathop \sum \limits_{{s = \left\{ {1, 2, \ldots ,K} \right\}}} \frac{{l_{s} }}{L}} \right) + 1} \right)$$

There is a noticeable difference in analyzing drug response from in vitro cancer cell lines and clinical cancer data. In clinical cancer data, drug response annotations are qualitative rather than quantitative. Drug responses are labeled as four types in TCGA: (1) complete response, (2) partial response, (3) clinical progressive disease, and (4) stable disease. We binarized such labels into “sensitive” and “resistant”. We considered drugs to be sensitive if annotations in TCGA were (1) complete response or (2) partial response. We considered drugs to be resistant if annotations were (3) clinical progressive disease or (4) stable disease. On the other hand, drug responses on cell lines are quantified by IC50. However, the range of IC50 of each drug is different (Figure S1 in Additional file 1). We thereby introduced a metric, drug sensitivity score (DSS), to transform drug responses into the same scale and to make responses comparable across drugs,

$$DSS = \left( { - 1} \right)^{I(IC50 > MSC)} ln\left( {\frac{{\left| {IC50 - MSC} \right|}}{MSC} + 1} \right)$$

where MSC denotes max screening concentration of the drug. We collected MSC from the GDSC. $I\left( \cdot \right)$ is indicator function. If $IC50 > MSC$, $I(IC50 > MSC) = 1$. This indicates that the given drug is not sufficient to kill the cancer cells, and the DSS is smaller than 0. If $IC50 < MSC$, $I(IC50 > MSC) = 0$. This indicates that the given drug has the potential to kill the cancer cells, and the DSS is larger than 0. The larger the DSS is, the more sensitive the drug is. Gene features and drug response annotations of clinical samples are given in Additional file 2: Table S8.

We predicted the IC50 of drugs on clinical cancer patients and calculated the DSS. We then adopted the ROC curve to analyze the consistency between our predictions and the binary clinical annotations obtained from the TCGA.

## Supplementary Information

**Additional file 1:** Supplementary figures and Supplementary tables S1–S7 for additional results. **Figure S1**. IC50 and MSC of drugs. **Figure S2**. PCA of structures of drugs. **Figure S3**. ROC curve on clinical cancer patients. **Table S1**. Parameter settings of DualGCN. **Table S2**. Descriptions of drugs. **Table S3**. Descriptions of cell lines. **Table S4**. List of cancer-related genes. **Table S5**. Results of SVM regression with various kernels. **Table S6**. Results of random forest with various number of trees. **Table S7**. Results of Lasso regression with various alpha.

**Additional file 2: Supplementary Table S8** for gene features and clinical annotations of the TCGA data.

## Data Availability

The source code is available at https://github.com/horsedayday/DualGCN.

## Abbreviations

SNV: Single nucleotide variant
CDR: Cancer drug response
IC50: Half-maximal inhibitory concentration
CCLE: Cancer Cell Line Encyclopedia
COSMIC: Catalogue of Somatic Mutations in Cancer
GDSC: Genomics of Drug Sensitivity in Cancer
SMILES: Simplified molecular-input line-entry system
MLP: Multilayer perceptron
CNN: Convolutional neural network
GCN: Graph convolutional network
PPI: Protein–protein interaction
Expr.: Gene expression
CNV: Copy number variation
SVM: Support vector machine
CV: Cross-validation
RMSE: Root mean square error
TCGA: The Cancer Genome Atlas Program
DSS: Drug sensitivity score
ROC: Receiver operating characteristic
AUC: Area under the curve
RBF: Radial basis function
