# Supplementary material for: DualGCN: a dual graph convolutional network model to predict cancer drug response
Source: BMC Bioinformatics. 2022 Apr 15;23(Suppl 4):129. doi: 10.1186/s12859-022-04664-4 (PMC9011932; doi:10.1186/s12859-022-04664-4)
Supplement: Supplementary file 1 — Additional file 1: Supplementary figures and Supplementary tables S1–S7 for additional results. Figure S1. IC50 and MSC of drugs. Figure S2. PCA of structures of drugs. Figure S3. ROC curve on clinical cancer patients. Table S1. Parameter settings of DualGCN. Table S2. Descriptions of drugs. Table S3. Descriptions of cell lines. Table S4. List of cancer-related genes. Table S5. Results of SVM regression with various kernels. Table S6. Results of random forest with various number of trees. Table S7. Results of Lasso regression with various alpha. [file 12859_2022_4664_MOESM1_ESM.pdf]

DualGCN: a dual graph convolutional network model to predict cancer drug response

Tianxing Ma[1], Qiao Liu[2], Haochen Li[3], Mu Zhou[4], Rui Jiang[1], and Xuegong Zhang[1,3,*]

[1] MOE Key Laboratory of Bioinformatics, Bioinformatics Division, BNRIST and Department of Automation, Tsinghua University, Beijing 100084, China
[2] Department of Statistics, Stanford University, Stanford, CA 94305
[3] School of Medicine, Center for Synthetic and Systems Biology, Tsinghua University, Beijing 100084, China
[4] SenseBrain Research, San Jose, CA 95131, USA
[*] Corresponding author: Xuegong Zhang, zhangxg@tsinghua.edu.cn

Contents

Figure S1: IC50 and MSC of drugs 2

Figure S2: PCA of structures of drugs 3

Figure S3: ROC curve on clinical cancer patients 4

Table S1: Parameter settings of DualGCN 5

Table S2: Descriptions of drugs 6

Table S3: Descriptions of cell lines 11

Table S4: List of cancer-related genes 23

Table S5: Results of SVM regression with various kernels 40

Table S6: Results of random forest with various number of trees 41

Table S7: Results of Lasso regression with various alpha 42

Figure S1: IC50 and MSC of drugs

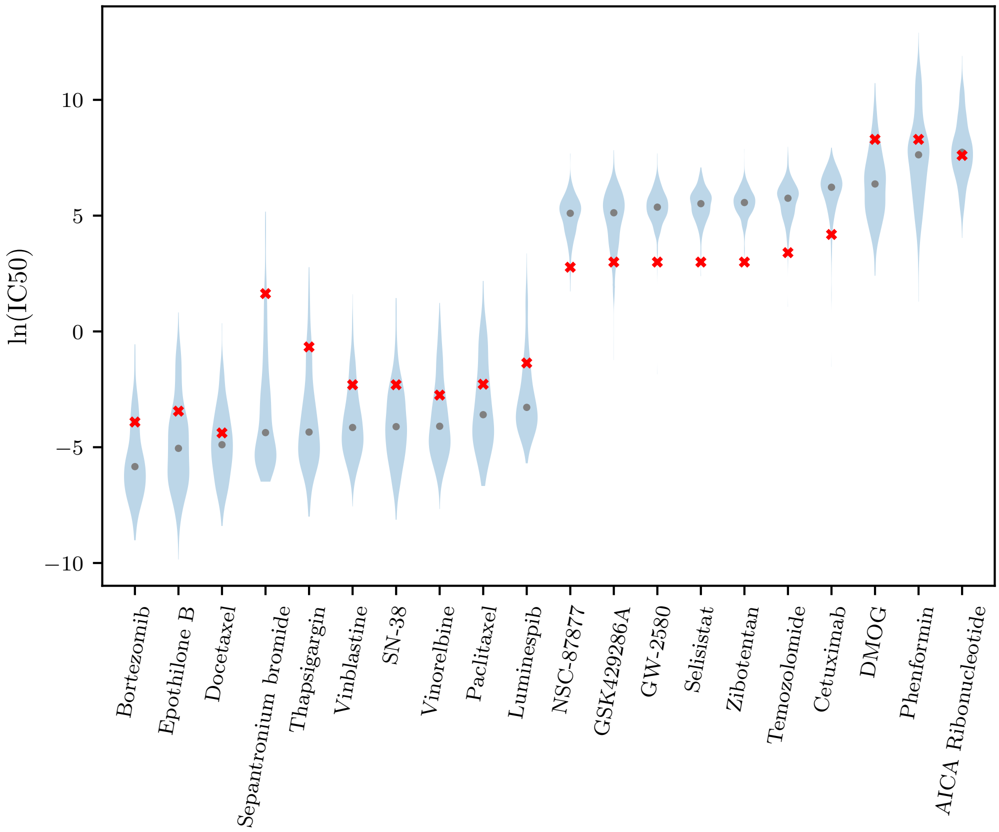

Figure S1 IC50 and max screening concentration (MSC) of drugs. We calculated the median of IC50 on each drug across all data and illustrated ten drugs with the smallest median (the left ten in the figure) and ten drugs with the largest median (the right ten in the figure). Blue violin plots describe IC50 among samples of related drugs. Gray points indicate the median. Red forks represent MSC.

Figure S2: PCA of structures of drugs

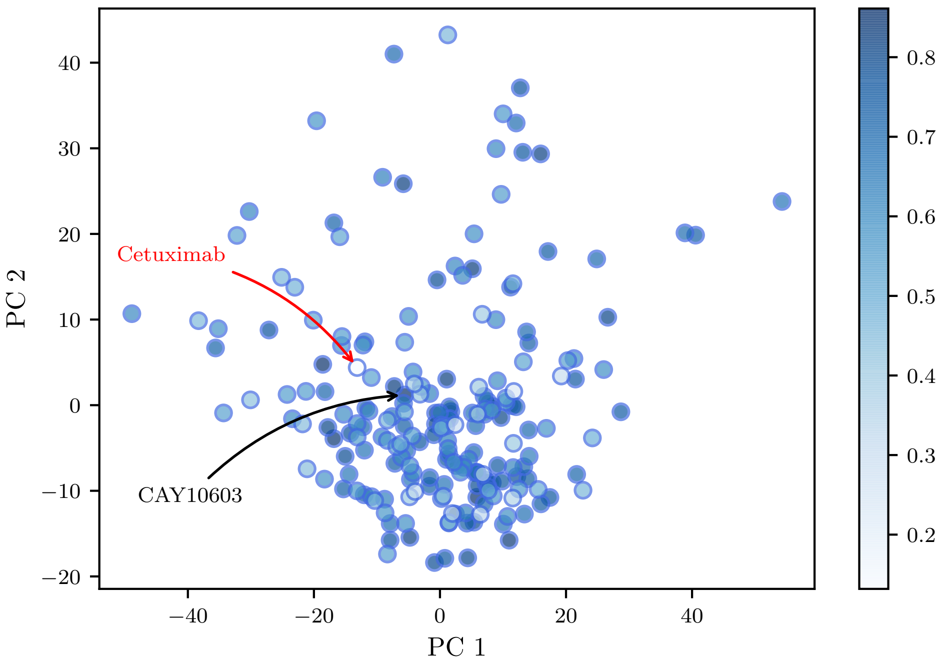

Figure S2 PCA of structures of drugs. We conducted PCA on SMILES of drugs. Each dot represents a drug. The degree of blue colors indicates average Pearson's correlation between the true and predicted IC50 of the drug. We marked the positions of cetuximab (with the worst predictive performance) and CAY10603 (with the best predictive performance) using red and black arrows, respectively.

Figure S3: ROC curve on clinical cancer patients

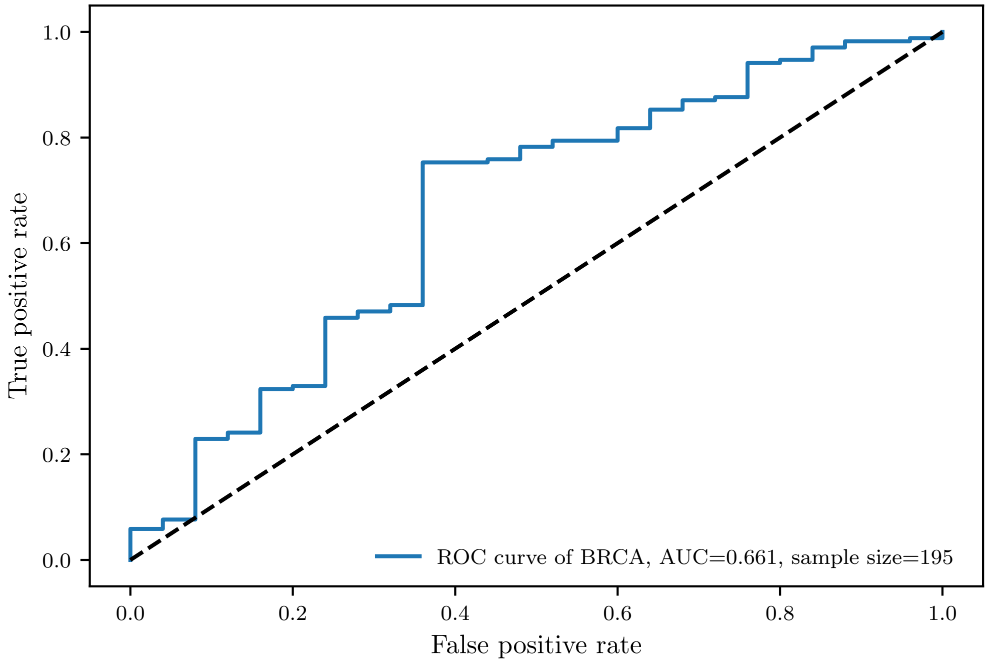

Figure S3 ROC curve on clinical cancer patients. We used the drug sensitivity score (DSS) as the discrimination threshold and varied the DSS to draw the ROC curve. The blue solid line represents the ROC curve, and the AUC of the ROC curve was 0.661.

Table S1: Parameter settings of DualGCN

Table S1 Parameter settings of DualGCN

	Operation	Output shape	Configuration
drug-GCN	features of drug atoms (input)	(None, 100, 75)	
	adjacency between atoms (input)	(None, 100, 100)	
	drug feature extraction (GCN #1)	(None, 100, 256)	activation: ReLU<br>batch normalization<br>dropout: dropout rate is 0.1
	drug feature extraction (GCN #2)	(None, 100, 128)	activation: ReLU<br>batch normalization<br>dropout: dropout rate is 0.1
	drug feature representation (pooling)	(None, 128)	global average pooling
bio-GCN	omics data of cancer samples (input)	(None, 697, 2)	
	adjacency between genes (input)	(None, 697, 697)	
	biological feature extraction (dense #1)	(None, 697, 32)	dropout: dropout rate is 0.1
	biological feature extraction (dense #2)	(None, 697, 128)	dropout: dropout rate is 0.1
	biological feature extraction (GCN #1)	(None, 697, 256)	activation: ReLU<br>batch normalization<br>dropout: dropout rate is 0.1
	biological feature extraction (GCN #2)	(None, 697, 256)	activation: ReLU<br>batch normalization<br>dropout: dropout rate is 0.1
	biological feature extraction (GCN #3)	(None, 697, 256)	activation: ReLU<br>batch normalization<br>dropout: dropout rate is 0.1
	biological feature extraction (GCN #4)	(None, 697, 256)	activation: ReLU<br>batch normalization<br>dropout: dropout rate is 0.1
	biological feature representation (pooling)	(None, 256)	global average pooling
drug-biosample	drug-biosample feature representation (concatenate)	(None, 384)	
MLP	drug response learning (dense #1)	(None, 256)	activation: tanh<br>dropout: dropout rate is 0.3
	drug response learning (dense #2)	(None, 128)	activation: tanh<br>dropout: dropout rate is 0.2
	drug response learning (dense #3)	(None, 10)	activation: tanh
	drug response learning (dense #4)	(None, 1)	

Table S2: Descriptions of drugs

Table S2 Descriptions of drugs

GDSC_id	PubChem_id	Name	Synonyms
1242	9863776	(5Z)-7-Oxozeaenol	5Z-7-Oxozeaenol, LL-Z1640-2
179	3385	5-Fluorouracil	5-FU
86	10172943	A-443654	KIN001-139
55	9549184	A-770041	KIN001-111
1001	65110	AICA Ribonucleotide	AICAR, N1-(b-D-Ribofuranosyl)-5-aminoimidazole-4-carboxamide
272	6918848	AR-42	HDAC-42, AR 42, AR42
207	10109823	AS601245	
224	5289247	AS605240	KIN001-173, AS-605240
219	11338033	AT-7519	AT7519
29	11676786	AZ628	AZ-628, AZ 628
1022	11152667	AZD7762	AZD-7762, AZD 7762
1059	25262965	AZD8055	AZD-8055
281	49806720	Alectinib	CH5424802, CH 542802, Alecensa
293	11282283	Amuvatinib	MP470, MP 470, MP-470
1021	6450551	Axitinib	AG-13736, Inlyta
178	10200390	BAY-61-3606	Syk Inhibitor, BAY-613606
60	11364421	BI-2536	
279	46931012	BIX02189	BIX 02189
203	9813758	BMS-345541	BMS345541, IKK Inhibitor 3
63	20635522	BMS-509744	KIN001-127, ITK inhibitor
184	24785538	BMS-754807	BMS754807, BMS 754807
222	11754511	BX-912	
1037	10077147	BX795	BX-795
274	6918638	Belinostat	PXD101, PXD-101
186	82146	Bexarotene	LG-100069, Targretin, Targret, Targrexin, Targretyn, Bexarotenum
1378	5460769	Bleomycin	
104	387447	Bortezomib	PS-341, LDP-341, Velcade
1019	5328940	Bosutinib	SKI-606, Bosulif
197	5280757	Bryostatin 1	Bryostatin
276	24951314	CAY10603	
1170	5327091	CCT-018159	CCT018159, CCT 018159
1067	2314623	CCT007093	
54	24825971	CGP-082996	CINK4, KIN001-021
53	644215	CGP-60474	KIN001-019, CGP60474, CGP 60474
1015	6918454	CI-1040	CI 1040, PD-18435, PD-184352, 212631-79-3
64	16663089	CMK	KIN001-128
152	44551660	CP466722	CP-466722, CP 466722, 1080622-86-1
255	9874913	CP724714	CP-724714
273	24756910	CUDC-101	CUDC 101
300	25257557	CX-5461	CX5461, CX 5461

249	25102847	Cabozantinib	BMS-907351, XL-184, Cometriq
1114	85668777	Cetuximab	Erbitux, IMC-C225, C225, IMC-225, L01XC06
1005	84691	Cisplatin	cis-Diammineplatinum(II) dichloride, Platinol, CIS-DDP
37	11626560	Crizotinib	Xalkori, PF2341066, PF-2341066, PF 2341066
1006	6253	Cytarabine	Ara-Cytidine, Arabinosyl Cytosine, U-19920
165	560326	DMOG	Dimethyloxalylglcine
1373	44462760	Dabrafenib	GSK2118436, Tafinlar
200	6445533	Dacinostat	NVP-LAQ824, LAQ824
1057	11977753	Dactolisib	NVP-BEZ235, BEZ235
1248	6914657	Daporinad	APO866, FK866, FK866
51	3062316	Dasatinib	BMS-354825-03, BMS-354825, Sprycel
1007	148124	Docetaxel	RP-56976, Taxotere
1042	156422	Doramapimod	BIRB-796, BIRB 796
133	31703	Doxorubicin	Doxil, Rubex, Adriamycin, Adriablastin, Doxorubicine
1069	9938202	EHT-1864	EHT 1864
1031	300471	Elesclomol	STA-4783
172	3218	Embelin	Emberine, Embelic acid
88	4261	Entinostat	MS-275
229	176167	Enzastaurin	LY317615
201	448013	Epothilone B	Patupilone, EpoB, EPO906, GNF-PF-193
1	176870	Erlotinib	Tarceva, RG-1415, CP-358774, OSI-774, Ro-508231, R-1415
134	36462	Etoposide	Etopophos, Vepesid, Eposin, VP-16
173	3463933	FH535	
263	11493598	FR-180204	FR 180204, FR180204, ERK Inhibitor II
166	3005532	FTI-277	
306	16722836	Fedratinib	TG101348, TG-101348, SAR302503, SAR-302503
308	42642645	Foretinib	GSK1363089, XL-880, EXEL-2880, GSK089
52	5311510	-GNF 2	KIN001-013
226	46885626	GSK1070916	GSK-1070916
202	25124816	GSK1904529A	GSK-1904529A, GSK 1904529A
91	11626927	GSK319347A	KIN001-135, IKK-3 inhibitor
230	11373846	GSK429286A	
177	25022668	GSK650394	GSK-650394, GSK 650394
326	16725726	GSK690693	GSK 690693, GSK-690693
193	11617559	GW-2580	GX2580, CFMS receptor tyrosine kinase inhibitor
1023	9943465	GW441756	GW 441756
87	9826308	GW843682X	GW843682X (AN-13)
1010	123631	Gefitinib	ZD-1839, Iressa
135	60750	Gemcitabine	Gemzar, LY-188011
159	53302361	HG6-64-1	KIN001-206
275	46943432	I-BET-762	GSK525762A
1230	54685215	IOX2	IOX-2, IOX 2, AK176060
176	521106	IPA-3	IPA 3
238	11625818	Idelalisib	CAL-101, Zydelig
34	5291	Imatinib	Gleevec, STI-571
208	6450816	Ispinesib Mesylate	SB-715992

1043	11624601	JNK Inhibitor VIII	
157	25222038	JNK-9L	KIN001-204, JNK inhibitor 9l
83	49836027	JW-7-52-1	
287	56965967	KIN001-244	PDK1 inhibitor 7
290	10451420	KIN001-260	Bayer IKKb inhibitor, ACHP
291	44143370	KIN001-266	
345	66577006	KIN001-270	CDK9 inhibitor, CDK9-IN-1
1030	5278396	KU-55933	KU55933
192	54676905	LFM-A13	DDE-28
119	208908	Lapatinib	Tykerb, Tyverb
1020	216326	Lenalidomide	CDC-501, CC-5013, Revlimid
1024	126565	Lestaurtinib	CEP-701, SP-924, SPM-924, A-154475, KT-555
277	11485656	Linifanib	ABT-869, ABT 869
185	11640390	Linsitinib	OSI-906, ASP-7487
194	10096043	Luminespib	AUY922, VER-52296,NVP-AUY922, AUY
9	462382	MG-132	LLL cpd, MG 132, MG132
1053	46930998	MK-2206	MK 2206, MK2206
294	25195352	MPS-1-IN-1	
292	10074640	Masitinib	AB1010, Masivet
1008	126941	Methotrexate	Abitrexate, Amethopterin, Rheumatrex, Trexall, Folex
136	5746	Mitomycin-C	Mytozytrex, NSC-26980, MMC, Mitosol, Mitozytrex
1029	11667893	Motesanib	AMG-706, AMG 706, AMG706
260	53340664	NG-25	NG25
269	42640	NSC-207895	XI-006, NSC207895
147	5459322	NSC-87877	NSC 87877
1038	11327430	NU7441	KU-57788, NU-7432, NU-7741
295	16747388	NVP-BHG712	BHG712
35	16038120	NVP-TAE684	NVP-TAE 684, TAE684, TAE-684
1011	24978538	Navitoclax	ABT-263, ABT263, ABT 263
1013	644241	Nilotinib	Tasigna, AMN 107
1047	11433190	Nutlin-3a (-)	
299	44224160	OSI-027	A-1065-5
298	9868037	OSI-930	OSI 930 OSI930
167	10027278	OSU-03012	AR-12, OSU 03012, OSU03012, PDK1 inhibitor AR-12
182	11404337	Obatoclax Mesylate	GX15-070MS, Obatoclax, GX15-070
283	25167777	Omipalisib	GSK2126458, GSK-2126458, EX-8678, GSK458
175	6753378	PAC-1	GTPL5238
1060	9826528	PD0325901	PD-0325901, PD 0325901
1049	1401	PD173074	PD-173074, PD 173074
1129	51371303	PF-4708671	PF 4708671, PF4708671
158	11713159	PF-562271	PF-00562271
1219	71271629	PFI-1	
1530	78243717	PFI-3	BDF00016096
6	10461815	PHA-665752	PHA665752, PHA 665752
301	46191454	PHA-793887	PHA793887, PHA 793887
302	9884685	PI-103	PI-103, PI103, PI 103

303	6852167	PIK-93	PIK 93, PIK93
11	36314	Paclitaxel	BMS-181339-01, Taxol, Onxol, Paxene, Praxel, Abraxane
1054	5330286	Palbociclib	PD0332991, PD-0332991, PF-00080665-73
89	7251185	Parthenolide	
199	10113978	Pazopanib	Votrient
282	6445562	Pelitinib	EKB-569, EKB 569
1529	16720766	Pevonedistat	MLN4924, MLN 4924, MLN-4924
196	8249	Phenformin	DBI
1243	637858	Piperlongumine	Piplartine
155	24826799	Ponatinib	AP24534, AP-24534, KIN001-192, Iclusig
71	4993	Pyrimethamine	Daraprim, Chloridine
151	4263900	QS11	944328-88-5
254	24889392	Quizartinib	AC220, AC 220, AC-220, Asp-2689
1052	44450571	RO-3306	
3	5384616	Rapamycin	AY-22989, Sirolimus, WY-090217, Torisel, Rapamune
1175	9931953	Rucaparib	PF-01367338, AG-014699, AG-14447, AG-14699
206	25126798	Ruxolitinib	INCB-18424, Ruxolitinib Phosphate, Jakafi
41	76044	S-Trityl-L-cysteine	NSC 83265, Tritylcysteine
1025	176158	SB216763	SB-216763, SB 216763
1194	9858940	SB505124	SB 505124, SB505124
304	9967941	SB52334	SB-52334, SB 52334
1061	11316960	SB590885	SB-590885
1264	56962337	SGC0946	
1039	10459196	SL0101	SL-0101, SL 0101-1
1494	104842	SN-38	7-Ethyl-10-Hydroxy-Camptothecin, SN 38
328	24772860	SNX-2112	SNX 2112
258	704473	STF-62247	STF62247
111	5717801	Salubrinal	EIF-2alpha Inhibitor
38	10302451	Saracatinib	AZD0530, AZD-0530, AZ-10353926
110	160355	Seliciclib	Roscovitine, CYC-202, AL-39256
341	5113032	Selisistat	EX-527, EX 527
268	11178236	Sepantronium bromide	YM155, YM-155, YM 155
1133	11609586	Serdemetan	JNJ-26854165
170	5208	Shikonin	Anchusin
30	216239	Sorafenib	Nexavar, 284461-73-0, BAY 43-9006
5	5329102	Sunitinib	Sutent, Sunitinib Malate, SU-11248
333	447912	T0901317	TO-901317, TO901317
221	9952773	TAK-715	KIN001-201, TAK 715
94	9907093	TGX221	TGX-221, Tgx 221
305	9903786	TPCA-1	
1149	11455910	TW 37	TW37, TW-37
1259	44819241	Talazoparib	BMN-673, BMN 973
1199	2733526	Tamoxifen	Nolvadex, Soltamox, Zynoplex, ICI-46474, Kessar
1026	6505803	Tanespimycin	17-AAG, BMS-722782
1375	5394	Temozolomide	Temodar, Temodal, M-39831, SCH 52365
1016	6918289	Temsirolimus	CCI-779, Torisel

180	446378	Thapsigargin	Octanoic acid
204	159324	Tipifarnib	Zarnestra
312	9911830	Tivozanib	AV-951, AV 951, KRN-951, KIL8951, ASP-4130
32	5494449	Tozasertib	MK 0457,MK-0457,MK-045, VX-680 VX 680 VX-68
1372	11707110	Trametinib	GSK1120212, Mekinist
1009	444795	Tretinoin	ATRA, Vesanoid, Renova, Atralin, Tretin-X, Avita
265	53394750	Tubastatin A	
1262	57339144	UNC1215	UNC-1215
271	24894414	VNLG/124	HDAC inhibitor XV
262	11634725	VX-11e	VX11e, VX11e
1028	10341154	VX-702	VX702, VX 702
1018	11960529	Veliparib	ABT-888, ABT888, ABT 888
1004	6710780	Vinblastine	Velban
140	5311497	Vinorelbine	vinorelbine tartrate, Navelbine, Exelbine
1033	24776445	Vismodegib	GDC0449, Erivedge
1012	5311	Vorinostat	Zolinza, SAHA, suberanilohydroxamic acid, suberoylanilide hydroxamic acid, MK-0683
56	11844351	WH-4-023	KIN001-112
288	3796	WHI-P97	AC1L1GQE, KIN001-055
59	49821040	WZ-1-84	KIN001-123
1046	10384072	Wee1 Inhibitor	681640, Wee1 Inhibitor
1268	2726824	XAV939	NVP-XAV939, XAV-939, XAV 939
106	46844147	XMD8-85	ERK5-IN-1
1164	46843772	XMD8-92	XMD 8-92
309	9810884	Y-39983	
1239	44632017	YK-4-279	YK 4-279
310	9956222	YM201636	YM-201636, YM 201636
45	16760646	Z-LLNle-CHO	Z-L-Norleucine-CHO, Gamma-Secretase Inhibitor 1
1050	9914412	ZM447439	ZM-447439, ZM 447439
223	11647372	ZSTK474	KIN001-167, ZSTK-474, ZSTK 474
266	9910224	Zibotentan	ZD4054, ZD-4054

Table S3: Descriptions of cell lines

Table S3 Descriptions of cell lines

DepMap ID	COSMIC ID	Sanger ID	CCLE name	Cancer type
ACH-000698	907295	580	DMS53_LUNG	SCLC
ACH-000489	909746	480	SW1116_LARGE_INTESTINE	COAD/READ
ACH-000522	724838	9	UMUC3_URINARY_TRACT	BLCA
ACH-000613	907060	1202	HOS_BONE	SARC
ACH-000614	909706	1274	RVH421_SKIN	SKCM
ACH-000691	749715	351	HCC2157_BREAST	BRCA
ACH-000610	688018	1220	NCIH2227_LUNG	SCLC
ACH-000420	909738	129	SNU449_LIVER	LIHC
ACH-000648	908470	1199	NCIH28_PLEURA	MESO
ACH-000091	1480362	686	OV56_OVARY	OV
ACH-000595	1240169	1504	LN229_CENTRAL_NERVOUS_SYSTEM	GBM
ACH-000770	909253	642	P31FUJ_HAEMATOPOIETIC_AND_LYMPHOID_TISSUE	LAML
ACH-000505	1298539	1471	RKN_SOFT_TISSUE	SARC
ACH-000609	909712	1677	SF126_CENTRAL_NERVOUS_SYSTEM	GBM
ACH-000974	909735	2058	SNGM_ENDOMETRIUM	UCEC
ACH-000875	687820	337	NCIH2347_LUNG	LUAD
ACH-000155	910907	1912	SW1990_PANCREAS	PAAD
ACH-000135	1298145	707	HS940T_FIBROBLAST	SKCM
ACH-000144	1240209	285	RERFGC1B_STOMACH	STAD
ACH-000054	907064	1391	HT1080_SOFT_TISSUE	SARC
ACH-000841	724834	915	NCIH2087_LUNG	LUAD
ACH-000451	687812	80	NCIH2085_LUNG	LUAD
ACH-000871	753605	919	NCIH510_LUNG	SCLC
ACH-000433	905963	289	CAKI1_KIDNEY	KIRC
ACH-000491	908458	649	NCIH716_LARGE_INTESTINE	COAD/READ
ACH-000727	1330973	1073	NCIH2066_LUNG	SCLC
ACH-000129	1330964	1825	NCIH1341_LUNG	SCLC
ACH-000298	688011	2106	NCIH2029_LUNG	SCLC
ACH-000616	1240151	1850	HS746T_STOMACH	STAD
ACH-000335	908152	209	MSTO211H_PLEURA	MESO
ACH-000654	909262	1762	RAJI_HAEMATOPOIETIC_AND_LYMPHOID_TISSUE	DLBC
ACH-000465	909723	42	SKMEL1_SKIN	SKCM
ACH-000953	909743	1452	SUPT1_HAEMATOPOIETIC_AND_LYMPHOID_TISSUE	ALL
ACH-000905	687452	1157	5637_URINARY_TRACT	BLCA
ACH-000258	906844	302	DU4475_BREAST	BRCA
ACH-000360	908442	35	NCIH508_LARGE_INTESTINE	COAD/READ
ACH-000893	910900	1828	NCIH1651_LUNG	LUAD
ACH-000939	909732	725	SKUT1_SOFT_TISSUE	SARC
ACH-000759	908120	50	MDAMB175VII_BREAST	BRCA
ACH-000059	1247871	786	SUPB15_HAEMATOPOIETIC_AND_LYMPHOID_TISSUE	ALL

ACH-000951	687819	1814	NCIH2342_LUNG	LUAD
ACH-000563	753554	1665	EBC1_LUNG	LUSC
ACH-000718	724874	917	NCIH2291_LUNG	LUAD
ACH-000147	905945	1286	T47D_BREAST	BRCA
ACH-000318	753622	1561	TE10_OESOPHAGUS	ESCA
ACH-000615	905954	1499	SKMEL28_SKIN	SKCM
ACH-000548	753535	748	BHY_UPPER_AERODIGESTIVE_TRACT	HNSC
ACH-000441	909713	1179	SH4_SKIN	SKCM
ACH-000501	907792	2266	LS123_LARGE_INTESTINE	COAD/READ
ACH-000004	907053	783	HEL_HAEMATOPOIETIC_AND_LYMPHOID_TISSUE	LAML
ACH-000270	1298136	744	HPAC_PANCREAS	PAAD
ACH-000189	909974	1640	RCC10RGB_KIDNEY	KIRC
ACH-000114	1240218	268	SU8686_PANCREAS	PAAD
ACH-000515	1303901	353	HCC33_LUNG	SCLC
ACH-000605	946355	1642	TE6_OESOPHAGUS	ESCA
ACH-000245	910706	737	BL41_HAEMATOPOIETIC_AND_LYMPHOID_TISSUE	DLBC
ACH-000161	906805	22	CORL105_LUNG	LUAD
ACH-000803	910692	1072	COLO668_LUNG	SCLC
ACH-000159	909250	1674	OSRC2_KIDNEY	KIRC
ACH-000042	1298475	1838	PANC0203_PANCREAS	PAAD
ACH-000794	1240121	1575	BICR22_UPPER_AERODIGESTIVE_TRACT	HNSC
ACH-000345	924189	852	KPNRTBM1_AUTONOMIC_GANGLIA	NB
ACH-000917	1503371	382	TE4_OESOPHAGUS	ESCA
ACH-000560	906848	799	ECC10_STOMACH	STAD
ACH-000305	753555	2138	ECGI10_OESOPHAGUS	ESCA
ACH-000998	910554	1833	CW2_LARGE_INTESTINE	COAD/READ
ACH-000532	1660035	151	SNU61_LARGE_INTESTINE	COAD/READ
ACH-000949	909770	1917	TGBC11TKB_STOMACH	STAD
ACH-000947	1480371	200	OVK18_OVARY	OV
ACH-000986	907289	2099	HT115_LARGE_INTESTINE	COAD/READ
ACH-000452	753623	2253	TE8_OESOPHAGUS	ESCA
ACH-000416	910399	484	NCIH838_LUNG	LUAD
ACH-000970	1674021	152	SNUC5_LARGE_INTESTINE	COAD/READ
ACH-000197	909762	2218	TALL1_HAEMATOPOIETIC_AND_LYMPHOID_TISSUE	ALL
ACH-000225	906849	2094	ECC12_STOMACH	STAD
ACH-000768	905960	1013	MDAMB231_BREAST	BRCA
ACH-000291	1240197	1327	OV90_OVARY	OV
ACH-000874	909703	862	RS411_HAEMATOPOIETIC_AND_LYMPHOID_TISSUE	ALL
ACH-000663	1298365	1095	OVTOKO_OVARY	OV
ACH-000617	905990	606	OVCAR4_OVARY	OV
ACH-000997	905937	1553	HCT15_LARGE_INTESTINE	COAD/READ
ACH-000470	909748	271	SW1463_LARGE_INTESTINE	COAD/READ
ACH-000376	905985	1164	SF295_CENTRAL_NERVOUS_SYSTEM	GBM
ACH-000304	909784	1000	WM115_SKIN	SKCM
ACH-000476	1240158	931	JHH4_LIVER	LIHC
ACH-000383	910549	1554	OE33_OESOPHAGUS	ESCA

ACH-000963	1240123	1915	CCK81_LARGE_INTESTINE	COAD/READ
ACH-000748	909717	1180	SJSA1_BONE	SARC
ACH-000447	687816	1812	NCIH2228_LUNG	LUAD
ACH-000665	909728	1628	SKMES1_LUNG	LUSC
ACH-000633	1290806	2062	FU97_STOMACH	STAD
ACH-000401	906813	21	COLO800_SKIN	SKCM
ACH-000729	688010	1570	NCIH1963_LUNG	SCLC
ACH-000965	930082	2215	RL952_ENDOMETRIUM	UCEC
ACH-000396	753566	1232	J82_URINARY_TRACT	BLCA
ACH-000878	1240143	1368	HCC15_LUNG	LUSC
ACH-000264	724859	1844	CALU6_LUNG	LUAD
ACH-000309	909721	233	SKLU1_LUNG	LUAD
ACH-000669	724879	1454	SW900_LUNG	LUSC
ACH-000425	905976	8	UACC62_SKIN	SKCM
ACH-000879	908130	920	MFE296_ENDOMETRIUM	UCEC
ACH-000756	906871	245	GI1_CENTRAL_NERVOUS_SYSTEM	LGG
ACH-000201	910784	349	A204_SOFT_TISSUE	SARC
ACH-000599	1298526	1549	PATU8902_PANCREAS	PAAD
ACH-000062	910931	2051	RERFLCMS_LUNG	LUAD
ACH-000243	1290797	871	DANG_PANCREAS	PAAD
ACH-000823	753573	951	KYSE140_OESOPHAGUS	ESCA
ACH-000693	907318	599	KYSE180_OESOPHAGUS	ESCA
ACH-000118	907285	109	HUPT3_PANCREAS	PAAD
ACH-000047	906869	1414	GCIY_STOMACH	STAD
ACH-000103	949090	1524	CAOV4_OVARY	OV
ACH-000755	749716	352	HCC2218_BREAST	BRCA
ACH-000351	908138	1647	MKN1_STOMACH	STAD
ACH-000472	753562	944	HSC2_UPPER_AERODIGESTIVE_TRACT	HNSC
ACH-000480	907071	2195	HUH7_LIVER	LIHC
ACH-000250	1298169	1515	KMRC20_KIDNEY	KIRC
ACH-000157	910934	186	A4FUK_HAEMATOPOIETIC_AND_LYMPHOID_TISSUE	DLBC
ACH-000231	907271	1936	KALS1_CENTRAL_NERVOUS_SYSTEM	LGG
ACH-000146	909771	770	THP1_HAEMATOPOIETIC_AND_LYMPHOID_TISSUE	LAML
ACH-000697	910935	1511	A3KAW_HAEMATOPOIETIC_AND_LYMPHOID_TISSUE	DLBC
ACH-000149	717431	1165	SKNSH_AUTONOMIC_GANGLIA	NB
ACH-000859	749709	2143	HCC1954_BREAST	BRCA
ACH-000783	946382	363	CAMA1_BREAST	BRCA
ACH-000269	910933	776	AM38_CENTRAL_NERVOUS_SYSTEM	GBM
ACH-000778	907061	648	HSC3_UPPER_AERODIGESTIVE_TRACT	HNSC
ACH-000546	907062	99	HSC4_UPPER_AERODIGESTIVE_TRACT	HNSC
ACH-000714	1659817	1308	KMS11_HAEMATOPOIETIC_AND_LYMPHOID_TISSUE	MM
ACH-000484	1240224	1459	VMRCRCW_KIDNEY	KIRC
ACH-000847	907055	1102	HGC27_STOMACH	STAD
ACH-000626	753615	1635	U266B1_HAEMATOPOIETIC_AND_LYMPHOID_TISSUE	MM
ACH-000674	1298357	941	NUGC4_STOMACH	STAD
ACH-000464	910943	604	CAS1_CENTRAL_NERVOUS_SYSTEM	GBM

ACH-000843	1240142	350	HARA_LUNG	LUSC
ACH-000811	905959	1166	SKOV3_OVARY	OV
ACH-000094	724869	889	HPAFII_PANCREAS	PAAD
ACH-000869	1298348	711	NCIH1568_LUNG	LUAD
ACH-000440	910703	1190	CA46_HAEMATOPOIETIC_AND_LYMPHOID_TISSUE	DLBC
ACH-000555	905948	602	A498_KIDNEY	KIRC
ACH-000052	684052	660	A673_BONE	SARC
ACH-000248	910704	726	AU565_BREAST	BRCA
ACH-000860	908465	1691	NCIH358_LUNG	LUAD
ACH-000138	906821	572	CFPAC1_PANCREAS	PAAD
ACH-000001	905933	2201	NIHOVCAR3_OVARY	OV
ACH-000035	687800	1548	NCIH1650_LUNG	LUAD
ACH-000888	908463	934	NCIH1793_LUNG	LUAD
ACH-000589	687794	800	NCIH1437_LUNG	LUAD
ACH-000858	753569	1369	KNS62_LUNG	LUSC
ACH-000148	905957	655	HS578T_BREAST	BRCA
ACH-000536	906801	2264	BT20_BREAST	BRCA
ACH-000483	1240216	1481	SNU182_LIVER	LIHC
ACH-000061	907056	310	HH_HAEMATOPOIETIC_AND_LYMPHOID_TISSUE	ALL
ACH-000846	906863	1170	FADU_UPPER_AERODIGESTIVE_TRACT	HNSC
ACH-000934	908121	880	MDAMB361_BREAST	BRCA
ACH-000573	1240172	927	MDAMB436_BREAST	BRCA
ACH-000334	906832	1185	DB_HAEMATOPOIETIC_AND_LYMPHOID_TISSUE	DLBC
ACH-000832	910916	1911	CAL27_UPPER_AERODIGESTIVE_TRACT	HNSC
ACH-000449	908127	1395	MESSA_SOFT_TISSUE	SARC
ACH-000890	1299062	2037	SW1271_LUNG	SCLC
ACH-000496	724868	2122	NCIH1792_LUNG	LUAD
ACH-000835	906999	963	GCT_SOFT_TISSUE	SARC
ACH-000355	688031	1545	NCIH82_LUNG	SCLC
ACH-000427	908461	88	NCIN87_STOMACH	STAD
ACH-000364	909776	1145	U2OS_BONE	SARC
ACH-000389	907042	1244	H4_CENTRAL_NERVOUS_SYSTEM	LGG
ACH-000081	906870	164	GDM1_HAEMATOPOIETIC_AND_LYMPHOID_TISSUE	LAML
ACH-000374	749710	1316	HCC1143_BREAST	BRCA
ACH-000018	724812	1455	T24_URINARY_TRACT	BLCA
ACH-000096	907299	962	G401_SOFT_TISSUE	SARC
ACH-000223	749714	936	HCC1937_BREAST	BRCA
ACH-000930	907046	2127	HCC1569_BREAST	BRCA
ACH-000247	1298358	1712	OCUM1_STOMACH	STAD
ACH-000410	909707	399	SAOS2_BONE	SARC
ACH-000181	909709	2054	SCC9_UPPER_AERODIGESTIVE_TRACT	HNSC
ACH-000699	749712	94	HCC1395_BREAST	BRCA
ACH-000200	908449	650	NMCG1_CENTRAL_NERVOUS_SYSTEM	LGG
ACH-000694	946353	1312	TE9_OESOPHAGUS	ESCA
ACH-000637	753575	894	KYSE520_OESOPHAGUS	ESCA
ACH-000469	909905	468	YH13_CENTRAL_NERVOUS_SYSTEM	GBM

ACH-000153	688058	1485	NCIH2052_PLEURA	MESO
ACH-000341	688087	227	SKNFI_AUTONOMIC_GANGLIA	NB
ACH-000260	724828	1480	SKNAS_AUTONOMIC_GANGLIA	NB
ACH-000162	1303896	1008	GA10_HAEMATOPOIETIC_AND_LYMPHOID_TISSUE	DLBC
ACH-000221	1240217	2169	SNU398_LIVER	LIHC
ACH-000493	909737	481	SNU423_LIVER	LIHC
ACH-000238	910904	1476	SCC4_UPPER_AERODIGESTIVE_TRACT	HNSC
ACH-000111	749711	1644	HCC1187_BREAST	BRCA
ACH-000478	909736	1482	SNU387_LIVER	LIHC
ACH-000786	906831	1783	DAUDI_HAEMATOPOIETIC_AND_LYMPHOID_TISSUE	DLBC
ACH-000914	907063	745	HT_HAEMATOPOIETIC_AND_LYMPHOID_TISSUE	DLBC
ACH-000151	1327768	2096	JM1_HAEMATOPOIETIC_AND_LYMPHOID_TISSUE	DLBC
ACH-000009	910700	2104	C2BBE1_LARGE_INTESTINE	COAD/READ
ACH-000087	684072	41	SKES1_BONE	SARC
ACH-000585	753556	404	EPLC272H_LUNG	LUSC
ACH-000535	906693	1978	BXPC3_PANCREAS	PAAD
ACH-000303	908445	28	SNU5_STOMACH	STAD
ACH-000422	909739	2059	SNU475_LIVER	LIHC
ACH-000348	910903	1909	RPMI7951_SKIN	SKCM
ACH-000812	1240125	857	COLO783_SKIN	SKCM
ACH-000228	1290725	940	BICR31_UPPER_AERODIGESTIVE_TRACT	HNSC
ACH-000968	906814	1467	COLO792_SKIN	SKCM
ACH-000007	907795	569	LS513_LARGE_INTESTINE	COAD/READ
ACH-000749	687985	1862	DMS273_LUNG	SCLC
ACH-000903	906864	1007	FTC133_THYROID	THCA
ACH-000187	687980	1167	CORL311_LUNG	SCLC
ACH-000827	1299081	114	WM793_SKIN	SKCM
ACH-000429	910920	949	A704_KIDNEY	KIRC
ACH-000987	908128	102	MEWO_SKIN	SKCM
ACH-000394	908480	914	NCIH2081_LUNG	SCLC
ACH-000514	687997	1928	NCIH1092_LUNG	SCLC
ACH-000421	909755	1141	SW837_LARGE_INTESTINE	COAD/READ
ACH-000882	907169	1534	IGR1_SKIN	SKCM
ACH-000780	908468	128	NCIH1105_LUNG	SCLC
ACH-000830	908469	2120	NCIH1436_LUNG	SCLC
ACH-000586	1330972	91	NCIH1876_LUNG	SCLC
ACH-000619	1240207	1810	PECAPJ15_UPPER_AERODIGESTIVE_TRACT	HNSC
ACH-000508	906808	774	CORL88_LUNG	SCLC
ACH-000743	1297439	1769	CORL95_LUNG	SCLC
ACH-000688	1480360	1985	OV7_OVARY	OV
ACH-000704	910548	782	OAW42_OVARY	OV
ACH-000935	1240173	928	MDST8_LARGE_INTESTINE	COAD/READ
ACH-000116	946360	173	OAW28_OVARY	OV
ACH-000652	1240219	1749	SUIT2_PANCREAS	PAAD
ACH-000361	909719	874	SKHEP1_LIVER	LIHC
ACH-000696	905991	2131	OVCAR8_OVARY	OV

ACH-000628	908459	1225	NCIH596_LUNG	LUAD
ACH-000945	722066	1539	NCIH650_LUNG	LUAD
ACH-000677	724878	849	SW1573_LUNG	LUAD
ACH-000400	909718	40	SKCO1_LARGE_INTESTINE	COAD/READ
ACH-000332	909904	895	YAPC_PANCREAS	PAAD
ACH-000547	907065	1533	HT1197_URINARY_TRACT	BLCA
ACH-000740	906794	2089	A253_SALIVARY_GLAND	HNSC
ACH-000244	906839	1386	DKMG_CENTRAL_NERVOUS_SYSTEM	GBM
ACH-000944	908159	669	NAMALWA_HAEMATOPOIETIC_AND_LYMPHOID_TISSUE	DLBC
ACH-000040	687588	7	U118MG_CENTRAL_NERVOUS_SYSTEM	GBM
ACH-000720	687459	275	TCCSUP_URINARY_TRACT	BLCA
ACH-000518	753541	2041	CAL33_UPPER_AERODIGESTIVE_TRACT	HNSC
ACH-000257	910937	1768	CORL279_LUNG	SCLC
ACH-000738	687568	2095	GB1_CENTRAL_NERVOUS_SYSTEM	GBM
ACH-000864	910691	218	COLO684_ENDOMETRIUM	UCEC
ACH-000991	1660036	697	SNU81_LARGE_INTESTINE	COAD/READ
ACH-000788	906792	652	A2058_SKIN	SKCM
ACH-000999	1659823	2191	SNU1040_LARGE_INTESTINE	COAD/READ
ACH-000989	1659928	154	SNU175_LARGE_INTESTINE	COAD/READ
ACH-000955	1660034	1907	SNU407_LARGE_INTESTINE	COAD/READ
ACH-000625	1240147	1922	HEP3B217_LIVER	LIHC
ACH-000607	1240166	1344	KYM1_SOFT_TISSUE	SARC
ACH-000641	910566	734	CMK_HAEMATOPOIETIC_AND_LYMPHOID_TISSUE	LAML
ACH-000656	1331038	1877	SUDHL8_HAEMATOPOIETIC_AND_LYMPHOID_TISSUE	DLBC
ACH-000227	907314	1977	KPNYN_AUTONOMIC_GANGLIA	NB
ACH-000329	906823	876	CCFSTTG1_CENTRAL_NERVOUS_SYSTEM	GBM
ACH-000050	724825	1230	NCIH929_HAEMATOPOIETIC_AND_LYMPHOID_TISSUE	MM
ACH-000781	1240187	1266	NCIH2023_LUNG	LUAD
ACH-000866	687995	1875	NCIH1048_LUNG	SCLC
ACH-000373	909722	304	SKM1_HAEMATOPOIETIC_AND_LYMPHOID_TISSUE	LAML
ACH-000322	907067	1193	HT144_SKIN	SKCM
ACH-000640	909727	153	SKMEL31_SKIN	SKCM
ACH-000814	1298144	1261	HS939T_SKIN	SKCM
ACH-000358	688027	1016	NCIH69_LUNG	SCLC
ACH-000386	907278	193	KG1_HAEMATOPOIETIC_AND_LYMPHOID_TISSUE	LAML
ACH-000668	907048	937	HCC70_BREAST	BRCA
ACH-000092	908462	624	NCIH2452_PLEURA	MESO
ACH-000932	908444	564	SNU1_STOMACH	STAD
ACH-000104	907789	1645	LOUCY_HAEMATOPOIETIC_AND_LYMPHOID_TISSUE	ALL
ACH-000467	1290907	1036	HCC56_LARGE_INTESTINE	COAD/READ
ACH-000766	687799	712	NCIH1648_LUNG	LUAD
ACH-000417	925347	635	PANC0813_PANCREAS	PAAD
ACH-000359	908131	2170	MG63_BONE	SARC
ACH-000900	905942	2108	NCIH23_LUNG	LUAD
ACH-000437	909745	1310	SW1088_CENTRAL_NERVOUS_SYSTEM	GBM
ACH-000744	687798	1238	NCIH1623_LUNG	LUAD

ACH-000733	687807	1690	NCIH1838_LUNG	LUAD
ACH-000559	1240182	1829	NCIH1836_LUNG	SCLC
ACH-000082	1290807	661	G292CLONEA141B1_BONE	SARC
ACH-000565	909263	1271	RCM1_LARGE_INTESTINE	COAD/READ
ACH-000008	910921	1806	A101D_SKIN	SKCM
ACH-000482	1298537	960	RERFLCKJ_LUNG	LUAD
ACH-000384	687457	2039	SW780_URINARY_TRACT	BLCA
ACH-000067	1240150	769	HS683_CENTRAL_NERVOUS_SYSTEM	LGG
ACH-000943	909698	607	RKO_LARGE_INTESTINE	COAD/READ
ACH-000132	1479995	436	JHOS2_OVARY	OV
ACH-000019	905946	588	MCF7_BREAST	BRCA
ACH-000725	1290906	1552	HCC202_BREAST	BRCA
ACH-000072	908126	1263	MEG01_HAEMATOPOIETIC_AND_LYMPHOID_TISSUE	LCML
ACH-000960	909696	1272	REH_HAEMATOPOIETIC_AND_LYMPHOID_TISSUE	ALL
ACH-000722	910905	1066	SNUC1_LARGE_INTESTINE	COAD/READ
ACH-000366	688086	867	SKNDZ_AUTONOMIC_GANGLIA	NB
ACH-000530	687983	1495	DMS114_LUNG	SCLC
ACH-000561	1299064	544	TT_OESOPHAGUS	ESCA
ACH-000567	910906	130	ST486_HAEMATOPOIETIC_AND_LYMPHOID_TISSUE	DLBC
ACH-000621	925338	1796	MDAMB157_BREAST	BRCA
ACH-000411	910922	1186	769P_KIDNEY	KIRC
ACH-000649	905947	301	786O_KIDNEY	KIRC
ACH-000713	906825	320	CAOV3_OVARY	OV
ACH-000816	908483	2147	NCIH524_LUNG	SCLC
ACH-000290	688013	452	NCIH209_LUNG	SCLC
ACH-000670	713880	999	SBC5_LUNG	SCLC
ACH-000644	687448	430	COLO829_SKIN	SKCM
ACH-000115	1299075	2154	VCAP_PROSTATE	PRAD
ACH-000915	907171	77	IPC298_SKIN	SKCM
ACH-000107	910915	703	CAPAN2_PANCREAS	PAAD
ACH-000117	1290798	1187	EFM192A_BREAST	BRCA
ACH-000178	1298141	1843	HS766T_PANCREAS	PAAD
ACH-000936	906852	1497	EFO27_OVARY	OV
ACH-000752	1240186	1250	NCIH196_LUNG	SCLC
ACH-000966	905968	1215	IGROV1_OVARY	OV
ACH-000639	1240189	2107	NCIH211_LUNG	SCLC
ACH-000822	909725	945	SKMEL24_SKIN	SKCM
ACH-000525	688015	1571	NCIH2171_LUNG	SCLC
ACH-000060	925348	197	PANC1005_PANCREAS	PAAD
ACH-000176	1298226	1506	LOUNH91_LUNG	LUSC
ACH-000885	1240222	700	TOV21G_OVARY	OV
ACH-000423	909724	1572	SKMEL3_SKIN	SKCM
ACH-000222	910702	59	ASPC1_PANCREAS	PAAD
ACH-000638	908460	1815	NCIH441_LUNG	LUAD
ACH-000521	722045	912	NCIH2030_LUNG	LUAD
ACH-000574	1240129	2063	FUOV1_OVARY	OV

ACH-000380	907281	2087	KMS12BM_HAEMATOPOIETIC_AND_LYMPHOID_TISSUE	MM
ACH-000002	905938	55	HL60_HAEMATOPOIETIC_AND_LYMPHOID_TISSUE	LAML
ACH-000863	906835	1784	DBTRG05MG_CENTRAL_NERVOUS_SYSTEM	GBM
ACH-000311	722046	1015	NCIH2122_LUNG	LUAD
ACH-000881	908125	626	MELJUSO_SKIN	SKCM
ACH-000651	905962	3	SW620_LARGE_INTESTINE	COAD/READ
ACH-000971	905936	1920	HCT116_LARGE_INTESTINE	COAD/READ
ACH-000171	909781	738	VMRCRCZ_KIDNEY	KIRC
ACH-000979	905935	872	DU145_PROSTATE	PRAD
ACH-000763	1659818	995	MM1S_HAEMATOPOIETIC_AND_LYMPHOID_TISSUE	MM
ACH-001190	905955	1191	SKMEL2_SKIN	SKCM
ACH-000681	905949	369	A549_LUNG	LUAD
ACH-000558	687563	1777	A172_CENTRAL_NERVOUS_SYSTEM	GBM
ACH-000587	924244	1403	NCIH1975_LUNG	LUAD
ACH-000805	906818	2049	COLO679_SKIN	SKCM
ACH-000475	1298146	1855	HUH1_LIVER	LIHC
ACH-000910	908122	101	MDAMB453_BREAST	BRCA
ACH-000235	1298476	2123	PANC0403_PANCREAS	PAAD
ACH-000527	1240198	358	OVISE_OVARY	OV
ACH-000330	906851	1786	EFM19_BREAST	BRCA
ACH-000450	908124	2109	MELHO_SKIN	SKCM
ACH-000371	910861	1273	RL_HAEMATOPOIETIC_AND_LYMPHOID_TISSUE	DLBC
ACH-000572	906865	1173	G361_SKIN	SKCM
ACH-000378	1240191	955	NCIH647_LUNG	LUAD
ACH-000102	906873	294	GMS10_CENTRAL_NERVOUS_SYSTEM	GBM
ACH-000323	687561	1465	42MGBA_CENTRAL_NERVOUS_SYSTEM	GBM
ACH-000667	1240145	1247	HCC44_LUNG	LUAD
ACH-000784	753576	2219	KYSE70_OESOPHAGUS	ESCA
ACH-000098	906868	1174	GAMG_CENTRAL_NERVOUS_SYSTEM	LGG
ACH-000343	905944	1224	NCIH522_LUNG	LUAD
ACH-000978	1240127	1919	EN_ENDOMETRIUM	UCEC
ACH-000985	907794	724	LS411N_LARGE_INTESTINE	COAD/READ
ACH-000562	1290908	639	HCC78_LUNG	LUAD
ACH-000570	687592	61	YKG1_CENTRAL_NERVOUS_SYSTEM	GBM
ACH-000913	907000	293	ESS1_ENDOMETRIUM	UCEC
ACH-000232	905983	1913	U251MG_CENTRAL_NERVOUS_SYSTEM	GBM
ACH-000488	946354	2073	TE11_OESOPHAGUS	ESCA
ACH-000647	753621	1493	TE1_OESOPHAGUS	ESCA
ACH-000408	735784	576	TE5_OESOPHAGUS	ESCA
ACH-000353	753614	575	TE15_OESOPHAGUS	ESCA
ACH-000528	906791	1557	ABC1_LUNG	LUAD
ACH-000137	687562	1207	8MGBA_CENTRAL_NERVOUS_SYSTEM	GBM
ACH-000880	906790	1011	AGS_STOMACH	STAD
ACH-000347	1298534	469	QGP1_PANCREAS	PAAD
ACH-000207	906837	2075	DETROIT562_UPPER_AERODIGESTIVE_TRACT	HNSC
ACH-000927	946359	412	BT474_BREAST	BRCA

ACH-000046	905950	371	ACHN_KIDNEY	KIRC
ACH-000950	907790	306	LOVO_LARGE_INTESTINE	COAD/READ
ACH-000810	909726	1789	SKMEL30_SKIN	SKCM
ACH-000443	1240199	1094	OVKATE_OVARY	OV
ACH-000956	924100	1027	22RV1_PROSTATE	PRAD
ACH-000580	906830	2242	C32_SKIN	SKCM
ACH-000139	925346	1873	PANC0327_PANCREAS	PAAD
ACH-000817	905964	705	RPMI8226_HAEMATOPOIETIC_AND_LYMPHOID_TISSUE	MM
ACH-000906	1240128	869	ES2_OVARY	OV
ACH-000327	684681	1826	NCIH1395_LUNG	LUAD
ACH-000379	1298350	2228	NCIH1781_LUNG	LUAD
ACH-000375	907298	1089	G402_SOFT_TISSUE	SARC
ACH-000506	910899	932	NCIH146_LUNG	SCLC
ACH-000958	909751	1	SW48_LARGE_INTESTINE	COAD/READ
ACH-000120	906820	13	CHP212_AUTONOMIC_GANGLIA	NB
ACH-000957	998189	2081	LS180_LARGE_INTESTINE	COAD/READ
ACH-000403	908457	573	NCIH747_LARGE_INTESTINE	COAD/READ
ACH-000242	687455	1309	RT4_URINARY_TRACT	BLCA
ACH-000793	907276	406	KATOIII_STOMACH	STAD
ACH-000236	909747	974	SW1417_LARGE_INTESTINE	COAD/READ
ACH-000680	909757	1689	SW948_LARGE_INTESTINE	COAD/READ
ACH-000876	924240	344	MDAMB415_BREAST	BRCA
ACH-000703	753551	300	DMS79_LUNG	SCLC
ACH-000849	908123	415	MDAMB468_BREAST	BRCA
ACH-000767	688025	1380	NCIH526_LUNG	SCLC
ACH-000048	1299070	5	TOV112D_OVARY	OV
ACH-000288	905951	1835	BT549_BREAST	BRCA
ACH-000399	908481	916	NCIH2196_LUNG	SCLC
ACH-000857	910852	113	CAL851_BREAST	BRCA
ACH-000308	911905	303	EFO21_OVARY	OV
ACH-000219	906793	953	A375_SKIN	SKCM
ACH-000800	688023	1222	NCIH446_LUNG	SCLC
ACH-000730	905956	1491	SKMEL5_SKIN	SKCM
ACH-000719	909699	1162	RMGI_OVARY	OV
ACH-000090	905934	911	PC3_PROSTATE	PRAD
ACH-000551	905940	2098	K562_HAEMATOPOIETIC_AND_LYMPHOID_TISSUE	LCML
ACH-000473	909704	25	RT112_URINARY_TRACT	BLCA
ACH-000911	908455	1251	NUGC3_STOMACH	STAD
ACH-000075	687590	549	U87MG_CENTRAL_NERVOUS_SYSTEM	GBM
ACH-000679	910079	1664	OE19_OESOPHAGUS	ESCA
ACH-000684	1298168	1514	KMRC1_KIDNEY	KIRC
ACH-000969	905989	1990	KM12_LARGE_INTESTINE	COAD/READ
ACH-000381	909761	997	T84_LARGE_INTESTINE	COAD/READ
ACH-000750	905974	401	LOXIMVI_SKIN	SKCM
ACH-000481	687815	1811	NCIH2170_LUNG	LUSC
ACH-000579	905977	14	UACC257_SKIN	SKCM

ACH-000824	907321	893	KYSE510_OESOPHAGUS	ESCA
ACH-000362	1330947	747	MOLM13_HAEMATOPOIETIC_AND_LYMPHOID_TISSUE	LAML
ACH-000855	907317	407	KYSE150_OESOPHAGUS	ESCA
ACH-000320	910546	560	PSN1_PANCREAS	PAAD
ACH-000023	1240201	1242	PATU8988T_PANCREAS	PAAD
ACH-000650	1240153	1535	IGR37_SKIN	SKCM
ACH-000188	910701	1419	SCC25_UPPER_AERODIGESTIVE_TRACT	HNSC
ACH-000078	908135	1818	MHHNB11_AUTONOMIC_GANGLIA	NB
ACH-000624	907047	1317	HCC1806_BREAST	BRCA
ACH-000073	1303897	1776	GRANTA519_HAEMATOPOIETIC_AND_LYMPHOID_TISSUE	DLBC
ACH-000006	908148	2167	MONOMAC6_HAEMATOPOIETIC_AND_LYMPHOID_TISSUE	LAML
ACH-000678	924250	2113	MKN7_STOMACH	STAD
ACH-000566	909749	2	SW1710_URINARY_TRACT	BLCA
ACH-000795	908146	86	MOLT13_HAEMATOPOIETIC_AND_LYMPHOID_TISSUE	ALL
ACH-000254	910911	2053	SCC15_UPPER_AERODIGESTIVE_TRACT	HNSC
ACH-000012	1240146	354	HCC827_LUNG	LUAD
ACH-000169	909264	1763	RD_SOFT_TISSUE	SARC
ACH-001306	906795	433	8305C_THYROID	THCA
ACH-000568	910910	1754	UACC812_BREAST	BRCA
ACH-000105	1295740	2263	ALLSIL_HAEMATOPOIETIC_AND_LYMPHOID_TISSUE	ALL
ACH-000981	1297446	1035	DND41_HAEMATOPOIETIC_AND_LYMPHOID_TISSUE	ALL
ACH-000439	1330942	427	ME1_HAEMATOPOIETIC_AND_LYMPHOID_TISSUE	LAML
ACH-000357	1327765	2118	JEKO1_HAEMATOPOIETIC_AND_LYMPHOID_TISSUE	DLBC
ACH-000798	1240124	555	CL40_LARGE_INTESTINE	COAD/READ
ACH-000349	1303900	653	HCC1500_BREAST	BRCA
ACH-000196	749713	1264	HCC1599_BREAST	BRCA
ACH-000336	1290455	355	OCIAML3_HAEMATOPOIETIC_AND_LYMPHOID_TISSUE	LAML
ACH-000271	1331033	269	SUDHL10_HAEMATOPOIETIC_AND_LYMPHOID_TISSUE	DLBC
ACH-000365	1331035	1450	SUDHL4_HAEMATOPOIETIC_AND_LYMPHOID_TISSUE	DLBC
ACH-000124	1330984	1972	OCILY19_HAEMATOPOIETIC_AND_LYMPHOID_TISSUE	DLBC
ACH-000363	753612	1003	SKMM2_HAEMATOPOIETIC_AND_LYMPHOID_TISSUE	MM
ACH-000554	909778	1090	UACC893_BREAST	BRCA
ACH-000136	910567	38	CHP126_AUTONOMIC_GANGLIA	NB
ACH-000717	906817	1469	COLO680N_OESOPHAGUS	ESCA
ACH-000350	910689	2148	COLO678_LARGE_INTESTINE	COAD/READ
ACH-000301	907783	709	LAMA84_HAEMATOPOIETIC_AND_LYMPHOID_TISSUE	LCML
ACH-000920	910951	160	CMLT1_HAEMATOPOIETIC_AND_LYMPHOID_TISSUE	LCML
ACH-000457	910952	482	CAL54_KIDNEY	KIRC
ACH-000432	910710	321	BV173_HAEMATOPOIETIC_AND_LYMPHOID_TISSUE	LCML
ACH-000056	906842	1339	DOHH2_HAEMATOPOIETIC_AND_LYMPHOID_TISSUE	DLBC
ACH-000198	906856	426	EOL1_HAEMATOPOIETIC_AND_LYMPHOID_TISSUE	LAML
ACH-000938	908158	973	NALM6_HAEMATOPOIETIC_AND_LYMPHOID_TISSUE	ALL
ACH-000545	909780	276	VMCUB1_URINARY_TRACT	BLCA
ACH-000204	907791	1794	LP1_HAEMATOPOIETIC_AND_LYMPHOID_TISSUE	MM
ACH-000787	753592	1175	LXF289_LUNG	LUAD
ACH-000218	1330991	698	PL21_HAEMATOPOIETIC_AND_LYMPHOID_TISSUE	LAML

ACH-000937	909260	1043	PF382_HAEMATOPOIETIC_AND_LYMPHOID_TISSUE	ALL
ACH-000113	910947	749	OCIAML2_HAEMATOPOIETIC_AND_LYMPHOID_TISSUE	LAML
ACH-000326	1327771	1807	JURLMK1_HAEMATOPOIETIC_AND_LYMPHOID_TISSUE	LCML
ACH-000065	1330983	2130	OCIAML5_HAEMATOPOIETIC_AND_LYMPHOID_TISSUE	LAML
ACH-000315	907274	1973	KARPAS422_HAEMATOPOIETIC_AND_LYMPHOID_TISSUE	DLBC
ACH-000643	1290922	743	HDQP1_BREAST	BRCA
ACH-000142	1290730	2040	CAL29_URINARY_TRACT	BLCA
ACH-001106	1330933	1206	KOPN8_HAEMATOPOIETIC_AND_LYMPHOID_TISSUE	ALL
ACH-000392	687777	219	CALU3_LUNG	LUAD
ACH-000852	1298347	2119	NCIH1435_LUNG	LUAD
ACH-000886	724873	79	NCIH2009_LUNG	LUAD
ACH-000277	907045	1551	HCC1419_BREAST	BRCA
ACH-000818	949093	1231	BT483_BREAST	BRCA
ACH-000352	1290905	1567	HCC1428_BREAST	BRCA
ACH-000074	907311	598	KU812_HAEMATOPOIETIC_AND_LYMPHOID_TISSUE	LCML
ACH-000790	724872	1479	SHP77_LUNG	SCLC
ACH-000772	924248	298	TE441T_SOFT_TISSUE	SARC
ACH-000804	949179	127	NB1_AUTONOMIC_GANGLIA	NB
ACH-000828	909907	429	ZR7530_BREAST	BRCA
ACH-000292	1240192	1372	NCIH841_LUNG	SCLC
ACH-000414	1240185	183	NCIH1944_LUNG	LUAD
ACH-000121	687821	82	NCIH2405_LUNG	LUAD
ACH-000636	909702	288	RPMI8402_HAEMATOPOIETIC_AND_LYMPHOID_TISSUE	ALL
ACH-000372	909251	2153	P12ICHIKAWA_HAEMATOPOIETIC_AND_LYMPHOID_TISSUE	ALL
ACH-000802	910926	1583	BFTC905_URINARY_TRACT	BLCA
ACH-000792	910698	1284	BFTC909_KIDNEY	KIRC
ACH-000168	908451	297	NOMO1_HAEMATOPOIETIC_AND_LYMPHOID_TISSUE	LAML
ACH-000356	925340	1542	MKN45_STOMACH	STAD
ACH-001307	924102	868	8505C_THYROID	THCA
ACH-000486	907312	892	KU1919_URINARY_TRACT	BLCA
ACH-000983	1330931	1513	KCL22_HAEMATOPOIETIC_AND_LYMPHOID_TISSUE	LCML
ACH-000210	753539	1523	CADOES1_BONE	SARC
ACH-000295	906855	901	EM2_HAEMATOPOIETIC_AND_LYMPHOID_TISSUE	LCML
ACH-000024	909249	112	OPM2_HAEMATOPOIETIC_AND_LYMPHOID_TISSUE	MM
ACH-000174	906828	1753	CAL62_THYROID	THCA
ACH-000995	998184	1210	JURKAT_HAEMATOPOIETIC_AND_LYMPHOID_TISSUE	ALL
ACH-000070	906800	63	697_HAEMATOPOIETIC_AND_LYMPHOID_TISSUE	ALL
ACH-000020	908132	1906	MHHCALL2_HAEMATOPOIETIC_AND_LYMPHOID_TISSUE	ALL
ACH-000918	908147	1321	MOLT16_HAEMATOPOIETIC_AND_LYMPHOID_TISSUE	ALL
ACH-000294	1323913	1285	NB4_HAEMATOPOIETIC_AND_LYMPHOID_TISSUE	LAML
ACH-000948	910924	558	2313287_STOMACH	STAD
ACH-000191	906696	672	BHT101_THYROID	THCA
ACH-000856	910927	4	CAL51_BREAST	BRCA
ACH-000183	924239	2224	L363_HAEMATOPOIETIC_AND_LYMPHOID_TISSUE	MM
ACH-000099	753620	2076	SIMA_AUTONOMIC_GANGLIA	NB
ACH-000287	1330982	95	NUDUL1_HAEMATOPOIETIC_AND_LYMPHOID_TISSUE	DLBC

ACH-000611	1331037	2036	SUDHL6_HAEMATOPOIETIC_AND_LYMPHOID_TISSUE	DLBC
ACH-000751	1330985	1679	OCIM1_HAEMATOPOIETIC_AND_LYMPHOID_TISSUE	LAML
ACH-000112	909715	656	SIGM5_HAEMATOPOIETIC_AND_LYMPHOID_TISSUE	LAML
ACH-000660	1331036	1609	SUDHL5_HAEMATOPOIETIC_AND_LYMPHOID_TISSUE	DLBC
ACH-000456	924104	346	BCPAP_THYROID	THCA
ACH-000973	906798	1483	639V_URINARY_TRACT	BLCA
ACH-000249	1290769	1153	CL11_LARGE_INTESTINE	COAD/READ
ACH-000716	1240223	1457	TT2609C02_THYROID	THCA
ACH-000895	1290771	1005	CL34_LARGE_INTESTINE	COAD/READ
ACH-000922	1330994	1160	RCHACV_HAEMATOPOIETIC_AND_LYMPHOID_TISSUE	ALL
ACH-000212	906826	1748	CAL120_BREAST	BRCA
ACH-000896	906797	2061	647V_URINARY_TRACT	BLCA
ACH-000689	971774	861	RH18_SOFT_TISSUE	SARC
ACH-000369	1330948	1229	MOLM16_HAEMATOPOIETIC_AND_LYMPHOID_TISSUE	LAML
ACH-000821	1297447	567	EJM_HAEMATOPOIETIC_AND_LYMPHOID_TISSUE	MM
ACH-000534	1331050	557	WSUDLCL2_HAEMATOPOIETIC_AND_LYMPHOID_TISSUE	DLBC
ACH-000058	1240178	1227	ML1_THYROID	THCA
ACH-000838	1295741	1204	AMO1_HAEMATOPOIETIC_AND_LYMPHOID_TISSUE	MM
ACH-000745	1330950	924	MOLP8_HAEMATOPOIETIC_AND_LYMPHOID_TISSUE	MM
ACH-000544	1298359	2152	OE21_OESOPHAGUS	ESCA
ACH-000861	905972	1500	HOP62_LUNG	LUAD
ACH-000706	905970	322	EKVX_LUNG	LUAD
ACH-000655	905986	1490	SF268_CENTRAL_NERVOUS_SYSTEM	GBM
ACH-000273	905984	1781	SF539_CENTRAL_NERVOUS_SYSTEM	LGG
ACH-000504	905982	1181	SNB75_CENTRAL_NERVOUS_SYSTEM	GBM

Table S4: List of cancer-related genes

Table S4 List of cancer-related genes

GeneSymbol	Name	Cytoband	GeneID
A1CF	APOBEC1 complementation factor	10q11.23	ENSG00000148584
ACVR2A	activin A receptor type 2A	2q22.3-q23.1	ENSG00000121989
AKT3	v-akt murine thymoma viral oncogene homolog 3	1q43-q44	ENSG00000117020
ANK1	ankyrin 1	8p11.21	ENSG00000029534
APOBEC3B	apolipoprotein B mRNA editing enzyme catalytic subunit 3B	22q13.1	ENSG00000179750
ARAF	A-Raf proto-oncogene, serine/threonine kinase	Xp11.3	ENSG00000078061
ARHGAP5	Rho GTPase activating protein 5	14q12	ENSG00000100852
ARHGEF10	Rho guanine nucleotide exchange factor 10	8p23.3	ENSG00000104728
ARHGEF10L	Rho guanine nucleotide exchange factor 10 like	1p36.13	ENSG00000074964
ASXL2	additional sex combs like 2, transcriptional regulator	2p23.3	ENSG00000143970
B2M	beta-2-microglobulin	15q21.1	ENSG00000166710
BARD1	BRCA1 associated RING domain 1	2q35	ENSG00000138376
BAX	BCL2 associated X, apoptosis regulator	19q13.33	ENSG00000087088
BAZ1A	bromodomain adjacent to zinc finger domain 1A	14q13.1-q13.2	ENSG00000198604
BCL2L12	BCL2 like 12	19q13.33	ENSG00000126453
BCL9L	B-cell CLL/lymphoma 9-like	11q23.3	ENSG00000186174
BCLAF1	BCL2 associated transcription factor 1	6q23.3	ENSG00000029363
BCORL1	BCL6 corepressor-like 1	Xq25-q26.1	ENSG00000085185
BIRC6	baculoviral IAP repeat containing 6	2p22.3	ENSG00000115760
BMP5	bone morphogenetic protein 5	6p12.1	ENSG00000112175
BTK	Bruton agammaglobulinemia tyrosine kinase	Xq21.33-q22	ENSG00000010671
CASP3	caspase 3	4q35.1	ENSG00000164305
CASP9	caspase 9	1p36.21	ENSG00000132906
CCNC	cyclin C	6q16.2	ENSG00000112237
CCR4	C-C motif chemokine receptor 4	3p22.3	ENSG00000183813
CCR7	C-C motif chemokine receptor 7	17q21.2	ENSG00000126353
CD209	CD209 molecule	19p13.2	ENSG00000090659
CD28	CD28 molecule	2q33.2	ENSG00000178562
CDH10	cadherin 10	5p14.2-p14.1	ENSG00000040731
CDH17	cadherin 17	8q22.1	ENSG00000079112
CDKN1A	cyclin dependent kinase inhibitor 1A	6p21.2	ENSG00000124762
CHST11	carbohydrate sulfotransferase 11	12q23.3	ENSG00000171310
CNBD1	cyclic nucleotide binding domain containing 1	8q21.3	ENSG00000176571
CNTNAP2	contactin associated protein like 2	7q35-q36.1	ENSG00000174469
COL3A1	collagen type III alpha 1 chain	2q32.2	ENSG00000168542
CPEB3	cytoplasmic polyadenylation element binding protein 3	10q23.32	ENSG00000107864
CRNKL1	crooked neck pre-mRNA splicing factor 1	20p11.23	ENSG00000101343
CSF1R	colony stimulating factor 1 receptor	5q32	ENSG00000182578
CSMD3	CUB and Sushi multiple domains 3	8q23.3	ENSG00000164796
CTNNA2	catenin alpha 2	2p12	ENSG00000066032

CTNND1	catenin delta 1	11q12.1	ENSG00000198561
CTNND2	catenin delta 2	5p15.2	ENSG00000169862
CUL3	cullin 3	2q36.2	ENSG00000036257
CYP2C8	cytochrome P450 family 2 subfamily C member 8	10q23.33	ENSG00000138115
CYSLTR2	cysteinyl leukotriene receptor 2	13q14.2	ENSG00000152207
DCAF12L2	DDB1 and CUL4 associated factor 12 like 2	Xq25	ENSG00000198354
DCC	DCC netrin 1 receptor	18q21.2	ENSG00000187323
DDR2	discoidin domain receptor 2	1q12-q23	ENSG00000162733
DDX3X	DEAD-box helicase 3, X-linked	Xp11.4	ENSG00000215301
DGCR8	DGCR8, microprocessor complex subunit	22q11.21	ENSG00000128191
DROSHA	drosha ribonuclease III	5q11.2	ENSG00000113360
EED	embryonic ectoderm development	11q14.2	ENSG00000074266
EIF1AX	eukaryotic translation initiation factor 1A, X-linked	Xp22.12	ENSG00000173674
ELF3	E74 like ETS transcription factor 3	1q32.1	ENSG00000163435
EPAS1	endothelial PAS domain protein 1	2p21-p16	ENSG00000116016
EPHA3	EPH receptor A3	3p11.1	ENSG00000044524
EPHA7	EPH receptor A7	6q16.1	ENSG00000135333
FAM135B	family with sequence similarity 135 member B	8q24.23	ENSG00000147724
FAM47C	family with sequence similarity 47 member C	Xp21.1	ENSG00000198173
FAT3	FAT atypical cadherin 3	11q14.3	ENSG00000165323
FBLN2	fibulin 2	3p25.1	ENSG00000163520
FEN1	flap structure-specific endonuclease 1	11q12.2	ENSG00000168496
FES	FES proto-oncogene, tyrosine kinase	15q26.1	ENSG00000182511
FKBP9	FK506 binding protein 9	7p14.3	ENSG00000122642
FLNA	filamin A	Xq28	ENSG00000196924
FOXR1	forkhead box R1	11q23.3	ENSG00000176302
GLI1	GLI family zinc finger 1	12q13.3	ENSG00000111087
GPC5	glypican 5	13q31.3	ENSG00000179399
GRM3	glutamate metabotropic receptor 3	7q21.11-q21.12	ENSG00000198822
ID3	inhibitor of DNA binding 3, HLH protein	1p36.12	ENSG00000117318
IGF2BP2	insulin like growth factor 2 mRNA binding protein 2	3q27.2	ENSG00000073792
IRS4	insulin receptor substrate 4	Xq22.3	ENSG00000133124
ISX	intestine specific homeobox	22q12.3	ENSG00000175329
ITGAV	integrin subunit alpha V	2q32.1	ENSG00000138448
KAT7	lysine acetyltransferase 7	17q21.33	ENSG00000136504
KEAP1	kelch like ECH associated protein 1	19p13.2	ENSG00000079999
KNSTRN	kinetochore localized astrin/SPAG5 binding protein	15q15.1	ENSG00000128944
LARP4B	La ribonucleoprotein domain family member 4B	10p15.3	ENSG00000107929
LATS1	large tumor suppressor kinase 1	6q25.1	ENSG00000131023
LATS2	large tumor suppressor kinase 2	13q12.11	ENSG00000150457
LEPROTL1	leptin receptor overlapping transcript like 1	8p12	ENSG00000104660
LRP1B	LDL receptor related protein 1B	2q21.2	ENSG00000168702
MACC1	MET transcriptional regulator MACC1	7p21.1	ENSG00000183742
MAPK1	mitogen-activated protein kinase 1	22q11.2	ENSG00000100030
MB21D2	Mab-21 domain containing 2	3q29	ENSG00000180611

MGMT	O-6-methylguanine-DNA methyltransferase	10q26.3	ENSG00000170430
MUC16	mucin 16, cell surface associated	19p13.2	ENSG00000181143
MUC4	mucin 4, cell surface associated	3q29	ENSG00000145113
N4BP2	NEDD4 binding protein 2	4p14	ENSG00000078177
NBEA	neurobeachin	13q13.3	ENSG00000172915
NTHL1	nth like DNA glycosylase 1	16p13.3	ENSG00000065057
PABPC1	poly(A) binding protein cytoplasmic 1	8q22.3	ENSG00000070756
PCBP1	poly(rC) binding protein 1	2p13.3	ENSG00000169564
PIK3CB	phosphatidylinositol-4,5-bisphosphate 3-kinase catalytic subunit beta	3q22.3	ENSG00000051382
POLD1	DNA polymerase delta 1, catalytic subunit	19q13.3	ENSG00000062822
POLG	DNA polymerase gamma, catalytic subunit	15q26.1	ENSG00000140521
POLQ	DNA polymerase theta	3q13.33	ENSG00000051341
PPM1D	protein phosphatase, Mg2+/Mn2+ dependent 1D	17q23.3	ENSG00000170836
PRDM2	PR/SET domain 2	1p36.21	ENSG00000116731
PREX2	phosphatidylinositol-3,4,5-trisphosphate dependent Rac exchange factor 2	8q13.2	ENSG00000046889
PRKCB	protein kinase C beta	16p12.2-p12.1	ENSG00000166501
PRPF40B	pre-mRNA processing factor 40 homolog B	12q13.12	ENSG00000110844
PTPN6	protein tyrosine phosphatase, non-receptor type 6	12p13.31	ENSG00000111679
PTPRD	protein tyrosine phosphatase, receptor type D	9p24.1-p23	ENSG00000153707
PTPRT	protein tyrosine phosphatase, receptor type T	20q12-q13	ENSG00000196090
QKI	QKI, KH domain containing, RNA binding	6q26	ENSG00000112531
RAD17	RAD17 checkpoint clamp loader component	5q13.2	ENSG00000152942
RFWD3	ring finger and WD repeat domain 3	16q23.1	ENSG00000168411
RGPD3	RANBP2-like and GRIP domain containing 3	2q12.2	ENSG00000153165
RGS7	regulator of G protein signaling 7	1q43	ENSG00000182901
ROBO2	roundabout guidance receptor 2	3p12.3	ENSG00000185008
S100A7	S100 calcium binding protein A7	1q21.3	ENSG00000143556
SALL4	spalt like transcription factor 4	20q13.2	ENSG00000101115
SETD1B	SET domain containing 1B	12q24.31	ENSG00000139718
SETDB1	SET domain bifurcated 1	1q21.3	ENSG00000143379
SFRP4	secreted frizzled related protein 4	7p14.1	ENSG00000106483
SGK1	serum/glucocorticoid regulated kinase 1	6q23.2	ENSG00000118515
SIRPA	signal regulatory protein alpha	20p13	ENSG00000198053
SIX1	SIX homeobox 1	14q23.1	ENSG00000126778
SIX2	SIX homeobox 2	2p21	ENSG00000170577
SKI	SKI proto-oncogene	1p36.33-p36.32	ENSG00000157933
SMC1A	structural maintenance of chromosomes 1A	Xp11.22	ENSG00000072501
SOX21	SRY-box 21	13q32.1	ENSG00000125285
SRC	SRC proto-oncogene, non-receptor tyrosine kinase	20q11.23	ENSG00000197122
STAG1	stromal antigen 1	3q22.3	ENSG00000118007
TAF15	TAF15 RNA polymerase II, TATA box binding protein (TBP)-associated factor, 68kDa	17q11.1-q11.2	ENSG00000172660
TEC	tec protein tyrosine kinase	4p12-p11	ENSG00000135605
TMEM127	transmembrane protein 127	2q11.2	ENSG00000135956

TNC	tenascin C	9q33.1	ENSG00000041982
TP63	tumor protein p63	3q28	ENSG00000073282
USP44	ubiquitin specific peptidase 44	12q21.33	ENSG00000136014
VAV1	vav guanine nucleotide exchange factor 1	19p13.3	ENSG00000141968
WNK2	WNK lysine deficient protein kinase 2	9q22.31	ENSG00000165238
ZEB1	zinc finger E-box binding homeobox 1	10p11.22	ENSG00000148516
ZMYM3	zinc finger MYM-type containing 3	Xq13.1	ENSG00000147130
ZNF429	zinc finger protein 429	19p12	ENSG00000197013
ZNF479	zinc finger protein 479	7p11.2	ENSG00000185177
ZNRF3	zinc and ring finger 3	22q12.1	ENSG00000183579
TP53	tumor protein p53	17p13.1	ENSG00000141510
PIK3CA	phosphatidylinositol-4,5-bisphosphate 3-kinase, catalytic subunit alpha	3q26.32	ENSG00000121879
FAT4	FAT atypical cadherin 4	4q28.1	ENSG00000196159
KMT2D	lysine (K)-specific methyltransferase 2D	12q13.12	ENSG00000167548
PTEN	phosphatase and tensin homolog	10q23.31	ENSG00000171862
KMT2C	lysine (K)-specific methyltransferase 2C	7q36.1	ENSG00000055609
ARID1A	AT rich interactive domain 1A (SWI-like)	1p36.11	ENSG00000117713
APC	adenomatous polyposis coli	5q22.2	ENSG00000134982
FAT1	FAT atypical cadherin 1	4q35.2	ENSG00000083857
KRAS	Kirsten rat sarcoma viral oncogene homolog	12p12.1	ENSG00000133703
BRAF	B-Raf proto-oncogene, serine/threonine kinase	7q34	ENSG00000157764
ATRX	alpha thalassemia/mental retardation syndrome X-linked	Xq21.1	ENSG00000085224
NF1	neurofibromin 1	17q11.2	ENSG00000196712
RNF213	ring finger protein 213	17q25.3	ENSG00000173821
ZFHX3	zinc finger homeobox 3	16q22.2, 16q22.3	ENSG00000140836
TRRAP	transformation/transcription domain-associated protein	7q22.1	ENSG00000196367
GRIN2A	glutamate receptor, ionotropic, N-methyl D-aspartate 2A	16p13.2	ENSG00000183454
ATM	ATM serine/threonine kinase	11q22.3	ENSG00000149311
AKAP9	A kinase (PRKA) anchor protein 9	7q21.2	ENSG00000127914
KMT2A	lysine (K)-specific methyltransferase 2A	11q23.3	ENSG00000118058
PTPRB	protein tyrosine phosphatase, receptor type, B	12q15	ENSG00000127329
SPEN	spen family transcriptional repressor	1p36.21, 1p36.13	ENSG00000065526
ERBB4	erb-b2 receptor tyrosine kinase 4	2q34	ENSG00000178568
CREBBP	CREB binding protein	16p13.3	ENSG00000005339
IDH1	isocitrate dehydrogenase 1 (NADP+), soluble	2q34	ENSG00000138413
FBXW7	F-box and WD repeat domain containing 7, E3 ubiquitin protein ligase	4q31.3	ENSG00000109670
ROS1	ROS proto-oncogene 1 , receptor tyrosine kinase	6q22.1	ENSG00000047936
EGFR	epidermal growth factor receptor	7p11.2	ENSG00000146648
PIK3R1	phosphoinositide-3-kinase, regulatory subunit 1 (alpha)	5q13.1	ENSG00000145675
RUNX1T1	runt-related transcription factor 1; translocated to, 1 (cyclin D-related)	8q21.3	ENSG00000079102
BRCA2	breast cancer 2, early onset	13q13.1	ENSG00000139618
MYH11	myosin, heavy chain 11, smooth muscle	16p13.11	ENSG00000133392
CDKN2A	cyclin-dependent kinase inhibitor 2A	9p21.3	ENSG00000147889

NOTCH1	notch 1	9q34.3	ENSG00000148400
ARID2	AT rich interactive domain 2 (ARID, RFX-like)	12q12	ENSG00000189079
EP300	E1A binding protein p300	22q13.2	ENSG00000100393
NCOR1	nuclear receptor corepressor 1	17p12, 17p11.2	ENSG00000141027
RANBP2	RAN binding protein 2	2q13	ENSG00000153201
RB1	retinoblastoma 1	13q14.2	ENSG00000139687
SMARCA4	SWI/SNF related, matrix associated, actin dependent regulator of chromatin, subfamily a, member 4	19p13.2	ENSG00000127616
PBRM1	polybromo 1	3p21.1	ENSG00000163939
NSD1	nuclear receptor binding SET domain protein 1	5q35.3	ENSG00000165671
CTNNB1	catenin (cadherin-associated protein), beta 1, 88kDa	3p22.1	ENSG00000168036
CHD4	chromodomain helicase DNA binding protein 4	12p13.31	ENSG00000111642
SETD2	SET domain containing 2	3p21.31	ENSG00000181555
ARID1B	AT rich interactive domain 1B (SWI1-like)	6q25.3	ENSG00000049618
ATR	ATR serine/threonine kinase	3q23	ENSG00000175054
UBR5	ubiquitin protein ligase E3 component n-recognin 5	8q22.3	ENSG00000104517
ZNF521	zinc finger protein 521	18q11.2	ENSG00000198795
MED12	mediator complex subunit 12	Xq13.1	ENSG00000184634
PDE4DIP	phosphodiesterase 4D interacting protein	1q21.2	ENSG00000178104
CAMTA1	calmodulin binding transcription activator 1	1p36.31, 1p36.23	ENSG00000171735
CDH11	cadherin 11, type 2, OB-cadherin (osteoblast)	16q21	ENSG00000140937
NCOR2	nuclear receptor corepressor 2	12q24.31	ENSG00000196498
TET1	tet methylcytosine dioxygenase 1	10q21.3	ENSG00000138336
CACNA1D	calcium channel, voltage-dependent, L type, alpha 1D subunit	3p21.1	ENSG00000157388
PTPRC	protein tyrosine phosphatase, receptor type, C	1q31.3, 1q32.1	ENSG00000081237
MYH9	myosin, heavy chain 9, non-muscle	22q12.3	ENSG00000100345
SETBP1	SET binding protein 1	18q12.3	ENSG00000152217
TPR	translocated promoter region, nuclear basket protein	1q31.1	ENSG00000047410
NTRK3	neurotrophic tyrosine kinase, receptor, type 3	15q25.3	ENSG00000140538
MTOR	mechanistic target of rapamycin (serine/threonine kinase)	1p36.22	ENSG00000198793
POLE	polymerase (DNA directed), epsilon, catalytic subunit	12q24.33	ENSG00000177084
CARD11	caspase recruitment domain family, member 11	7p22.2	ENSG00000198286
AMER1	APC membrane recruitment protein 1	Xq11.2	ENSG00000184675
KDR	kinase insert domain receptor	4q12	ENSG00000128052
PDGFRA	platelet-derived growth factor receptor, alpha polypeptide	4q12	ENSG00000134853
TET2	tet methylcytosine dioxygenase 2	4q24	ENSG00000168769
BCL11A	B-cell CLL/lymphoma 11A (zinc finger protein)	2p16.1	ENSG00000119866
ALK	anaplastic lymphoma receptor tyrosine kinase	2p23.2, 2p23.1	ENSG00000171094
KDM6A	lysine (K)-specific demethylase 6A	Xp11.3	ENSG00000147050
BCOR	BCL6 corepressor	Xp11.4	ENSG00000183337
MECOM	MDS1 and EVI1 complex locus	3q26.2	ENSG00000085276
CIC	capicua transcriptional repressor	19q13.2	ENSG00000079432
FLT4	fms-related tyrosine kinase 4	5q35.3	ENSG00000037280
KIAA1549	KIAA1549	7q34	ENSG00000122778

AFF3	AF4/FMR2 family, member 3	2q11.2	ENSG00000144218
CUX1	cut-like homeobox 1	7q22.1	ENSG00000257923
ERBB3	erb-b2 receptor tyrosine kinase 3	12q13.2	ENSG00000065361
NOTCH2	notch 2	1p12	ENSG00000134250
PTPRK	protein tyrosine phosphatase, receptor type, K	6q22.33	ENSG00000152894
KAT6A	K(lysine) acetyltransferase 6A	8p11.21	ENSG00000083168
ATP2B3	ATPase, Ca++ transporting, plasma membrane 3	Xq28	ENSG00000067842
CDH1	cadherin 1, type 1, E-cadherin (epithelial)	16q22.1	ENSG00000039068
STAG2	stromal antigen 2	Xq25	ENSG00000101972
PTPN13	protein tyrosine phosphatase, non-receptor type 13 (APO-1/CD95 (Fas)-associated phosphatase)	4q21.3	ENSG00000163629
NRG1	neuregulin 1	8p12	ENSG00000157168
NFE2L2	nuclear factor, erythroid 2-like 2	2q31.2	ENSG00000116044
PTCH1	patched 1	9q22.32	ENSG00000185920
KDM5A	lysine (K)-specific demethylase 5A	12p13.33	ENSG00000073614
NIN	ninein (GSK3B interacting protein)	14q22.1	ENSG00000100503
GATA3	GATA binding protein 3	10p14	ENSG00000107485
ERBB2	erb-b2 receptor tyrosine kinase 2	17q12	ENSG00000141736
GNAS	GNAS complex locus	20q13.32	ENSG00000087460
SMAD4	SMAD family member 4	18q21.2	ENSG00000141646
DICER1	dicer 1, ribonuclease type III	14q32.13	ENSG00000100697
ASXL1	additional sex combs like transcriptional regulator 1	20q11.21	ENSG00000171456
COL2A1	collagen, type II, alpha 1	12q13.11	ENSG00000139219
USP6	ubiquitin specific peptidase 6	17p13.2	ENSG00000129204
BCL11B	B-cell CLL/lymphoma 11B (zinc finger protein)	14q32.2	ENSG00000127152
RET	ret proto-oncogene	10q11.21	ENSG00000165731
PRDM16	PR domain containing 16	1p36.32	ENSG00000142611
CTCF	CCCTC-binding factor (zinc finger protein)	16q22.1	ENSG00000102974
NUMA1	nuclear mitotic apparatus protein 1	11q13.4	ENSG00000137497
KIT	v-kit Hardy-Zuckerman 4 feline sarcoma viral oncogene homolog	4q12	ENSG00000157404
BRCA1	breast cancer 1, early onset	17q21.31	ENSG00000012048
CDK12	cyclin-dependent kinase 12	17q12	ENSG00000167258
EBF1	early B-cell factor 1	5q33.3	ENSG00000164330
BCL9	B-cell CLL/lymphoma 9	1q21.2	ENSG00000116128
DNMT3A	DNA (cytosine-5-)-methyltransferase 3 alpha	2p23.3	ENSG00000119772
NACA	nascent polypeptide-associated complex alpha subunit	12q13.3	ENSG00000196531
BCR	breakpoint cluster region	22q11.23	ENSG00000186716
MAP3K1	mitogen-activated protein kinase kinase kinase 1, E3 ubiquitin protein ligase	5q11.2	ENSG00000095015
RNF43	ring finger protein 43	17q22	ENSG00000108375
TSC2	tuberous sclerosis 2	16p13.3	ENSG00000103197
KAT6B	K(lysine) acetyltransferase 6B	10q22.2	ENSG00000156650
NRAS	neuroblastoma RAS viral (v-ras) oncogene homolog	1p13.2	ENSG00000213281
CNTRL	centriolin	9q33.2	ENSG00000119397
MYO5A	myosin VA (heavy chain 12, myoxin)	15q21.2	ENSG00000197535
COL1A1	collagen, type I, alpha 1	17q21.33	ENSG00000108821

SF3B1	splicing factor 3b, subunit 1, 155kDa	2q33.1	ENSG00000115524
FGFR2	fibroblast growth factor receptor 2	10q26.13	ENSG00000066468
NUP214	nucleoporin 214kDa	9q34.13	ENSG00000126883
NUP98	nucleoporin 98kDa	11p15.4	ENSG00000110713
FLT3	fms-related tyrosine kinase 3	13q12.2	ENSG00000122025
PAX3	paired box 3	2q36.1	ENSG00000135903
MET	MET proto-oncogene, receptor tyrosine kinase	7q31.2	ENSG00000105976
IL7R	interleukin 7 receptor	5p13.2	ENSG00000168685
JAK1	Janus kinase 1	1p31.3	ENSG00000162434
LIFR	leukemia inhibitory factor receptor alpha	5p13.1	ENSG00000113594
FANCD2	Fanconi anemia, complementation group D2	3p25.3	ENSG00000144554
SRGAP3	SLIT-ROBO Rho GTPase activating protein 3	3p25.3	ENSG00000196220
BRD4	bromodomain containing 4	19p13.12	ENSG00000141867
BAP1	BRCA1 associated protein-1 (ubiquitin carboxy-terminal hydrolase)	3p21.1	ENSG00000163930
NFATC2	nuclear factor of activated T-cells, cytoplasmic, calcineurin-dependent 2	20q13.2	ENSG00000101096
AR	androgen receptor	Xq12	ENSG00000169083
CASP8	caspase 8, apoptosis-related cysteine peptidase	2q33.1	ENSG00000064012
NUTM1	NUT midline carcinoma, family member 1	15q14	ENSG00000184507
ABL2	ABL proto-oncogene 2, non-receptor tyrosine kinase	1q25.2	ENSG00000143322
CLTCL1	clathrin, heavy chain-like 1	22q11.21	ENSG00000070371
AFF1	AF4/FMR2 family, member 1	4q21.3, 4q22.1	ENSG00000172493
CIITA	class II, major histocompatibility complex, transactivator	16p13.13	ENSG00000179583
KDM5C	lysine (K)-specific demethylase 5C	Xp11.22	ENSG00000126012
JAK3	Janus kinase 3	19p13.11	ENSG00000105639
FANCA	Fanconi anemia, complementation group A	16q24.3	ENSG00000187741
TSC1	tuberous sclerosis 1	9q34.13	ENSG00000165699
WT1	Wilms tumor 1	11p13	ENSG00000184937
DCTN1	dynactin 1	2p13.1	ENSG00000204843
CLTC	clathrin, heavy chain (Hc)	17q23.1	ENSG00000141367
FOXP1	forkhead box P1	3p13	ENSG00000114861
RANBP17	RAN binding protein 17	5q35.1	ENSG00000204764
CBLB	Cbl proto-oncogene B, E3 ubiquitin protein ligase	3q13.11	ENSG00000114423
NCOA1	nuclear receptor coactivator 1	2p23.3	ENSG00000084676
PRDM1	PR domain containing 1, with ZNF domain	6q21	ENSG00000057657
SND1	staphylococcal nuclease and tudor domain containing 1	7q32.1	ENSG00000197157
TRIP11	thyroid hormone receptor interactor 11	14q32.12	ENSG00000100815
NCOA2	nuclear receptor coactivator 2	8q13.3	ENSG00000140396
LZTR1	leucine-zipper-like transcription regulator 1	22q11.21	ENSG00000099949
ARHGEF12	Rho guanine nucleotide exchange factor (GEF) 12	11q23.3	ENSG00000196914
PDGFRB	platelet-derived growth factor receptor, beta polypeptide	5q32	ENSG00000113721
PML	promyelocytic leukemia	15q24.1	ENSG00000140464
SPECC1	sperm antigen with calponin homology and coiled-coil domains 1	17p11.2	ENSG00000128487
PPP2R1A	protein phosphatase 2, regulatory subunit A, alpha	19q13.41	ENSG00000105568
TBX3	T-box 3	12q24.21	ENSG00000135111

CLIP1	CAP-GLY domain containing linker protein 1	12q24.31	ENSG00000130779
IKZF1	IKAROS family zinc finger 1 (Ikaros)	7p12.2	ENSG00000185811
BRIP1	BRCA1 interacting protein C-terminal helicase 1	17q23.2	ENSG00000136492
DNM2	dynamin 2	19p13.2	ENSG00000079805
WRN	Werner syndrome, RecQ helicase-like	8p12	ENSG00000165392
MSH6	mutS homolog 6	2p16.3	ENSG00000116062
AFF4	AF4/FMR2 family, member 4	5q31.1	ENSG00000072364
PLAG1	pleiomorphic adenoma gene 1	8q12.1	ENSG00000181690
NTRK1	neurotrophic tyrosine kinase, receptor, type 1	1q23.1	ENSG00000198400
RBM10	RNA binding motif protein 10	Xp11.3	ENSG00000182872
KTN1	kinectin 1 (kinesin receptor)	14q22.3	ENSG00000126777
AXIN1	axin 1	16p13.3	ENSG00000103126
ABL1	ABL proto-oncogene 1, non-receptor tyrosine kinase	9q34.12	ENSG00000097007
MAML2	mastermind-like 2 (Drosophila)	11q21	ENSG00000184384
MAP3K13	mitogen-activated protein kinase kinase kinase 13	3q27.2	ENSG00000073803
ERCC5	excision repair cross-complementation group 5	13q33.1	ENSG00000134899
MN1	meningioma (disrupted in balanced translocation) 1	22q12.1	ENSG00000169184
ZMYM2	zinc finger, MYM-type 2	13q12.11	ENSG00000121741
AXIN2	axin 2	17q24.1	ENSG00000168646
CYLD	cylindromatosis (turban tumor syndrome)	16q12.1	ENSG00000083799
TCF7L2	transcription factor 7-like 2 (T-cell specific, HMG-box)	10q25.2, 10q25.3	ENSG00000148737
FCRL4	Fc receptor-like 4	1q23.1	ENSG00000163518
TSHR	thyroid stimulating hormone receptor	14q31.1	ENSG00000165409
PLCG1	phospholipase C, gamma 1	20q12	ENSG00000124181
SLC34A2	solute carrier family 34 (type II sodium/phosphate cotransporter), member 2	4p15.2	ENSG00000157765
PER1	period circadian clock 1	17p13.1	ENSG00000179094
BLM	Bloom syndrome, RecQ helicase-like	15q26.1	ENSG00000197299
VHL	von Hippel-Lindau tumor suppressor, E3 ubiquitin protein ligase	3p25.3	ENSG00000134086
RUNX1	runt-related transcription factor 1	21q22.12	ENSG00000159216
PCM1	pericentriolar material 1	8p22	ENSG00000078674
ERCC2	excision repair cross-complementation group 2	19q13.32	ENSG00000104884
TGFBR2	transforming growth factor, beta receptor II (70/80kDa)	3p24.1	ENSG00000163513
EIF4A2	eukaryotic translation initiation factor 4A2	3q27.3	ENSG00000156976
NFIB	nuclear factor I/B	9p23, 9p22.3	ENSG00000147862
ZBTB16	zinc finger and BTB domain containing 16	11q23.2	ENSG00000109906
FGFR3	fibroblast growth factor receptor 3	4p16.3	ENSG00000068078
CNOT3	CCR4-NOT transcription complex, subunit 3	19q13.42	ENSG00000088038
SPOP	speckle-type POZ protein	17q21.33	ENSG00000121067
HIP1	huntingtin interacting protein 1	7q11.23	ENSG00000127946
ELN	elastin	7q11.23	ENSG00000049540
ESR1	estrogen receptor 1	6q25.1, 6q25.2	ENSG00000091831
THRAP3	thyroid hormone receptor associated protein 3	1p34.3	ENSG00000054118
STAT3	signal transducer and activator of transcription 3 (acute-phase response factor)	17q21.2	ENSG00000168610

JAK2	Janus kinase 2	9p24.1	ENSG00000096968
LRIG3	leucine-rich repeats and immunoglobulin-like domains 3	12q14.1	ENSG00000139263
FGFR1	fibroblast growth factor receptor 1	8p11.23	ENSG00000077782
STIL	SCL/TAL1 interrupting locus	1p33	ENSG00000123473
ITK	IL2-inducible T-cell kinase	5q33.3	ENSG00000113263
EML4	echinoderm microtubule associated protein like 4	2p21	ENSG00000143924
FGFR4	fibroblast growth factor receptor 4	5q35.2	ENSG00000160867
TRIM33	tripartite motif containing 33	1p13.2	ENSG00000197323
ATP1A1	ATPase, Na+/K+ transporting, alpha 1 polypeptide	1p13.1	ENSG00000163399
IL21R	interleukin 21 receptor	16p12.1	ENSG00000103522
PMS2	PMS2 postmeiotic segregation increased 2 (S. cerevisiae)	7p22.1	ENSG00000122512
XPO1	exportin 1	2p15	ENSG00000082898
MLLT10	myeloid/lymphoid or mixed-lineage leukemia (trithorax homolog, Drosophila); translocated to, 10	10p12.31	ENSG00000078403
ERG	v-ets avian erythroblastosis virus E26 oncogene homolog	21q22.2	ENSG00000157554
IKBKB	inhibitor of kappa light polypeptide gene enhancer in B-cells, kinase beta	8p11.21	ENSG00000104365
PALB2	partner and localizer of BRCA2	16p12.2	ENSG00000083093
BRD3	bromodomain containing 3	9q34.2	ENSG00000169925
RALGDS	ral guanine nucleotide dissociation stimulator	9q34.13, 9q34.2	ENSG00000160271
CBL	Cbl proto-oncogene, E3 ubiquitin protein ligase	11q23.3	ENSG00000110395
FLI1	Fli-1 proto-oncogene, ETS transcription factor	11q24.3	ENSG00000151702
ERCC3	excision repair cross-complementation group 3	2q14.3	ENSG00000163161
LPP	LIM domain containing preferred translocation partner in lipoma	3q27.3, 3q28	ENSG00000145012
ZNF331	zinc finger protein 331	19q13.42	ENSG00000130844
MAP2K4	mitogen-activated protein kinase kinase 4	17p12	ENSG00000065559
ERC1	ELKS/RAB6-interacting/CAST family member 1	12p13.33	ENSG00000082805
MLLT3	myeloid/lymphoid or mixed-lineage leukemia (trithorax homolog, Drosophila); translocated to, 3	9p21.3	ENSG00000171843
ETV1	ets variant 1	7p21.2	ENSG00000006468
FOXA1	forkhead box A1	14q21.1	ENSG00000129514
CSF3R	colony stimulating factor 3 receptor (granulocyte)	1p34.3	ENSG00000119535
GPHN	gephyrin	14q23.3	ENSG00000171723
ERCC4	excision repair cross-complementation group 4	16p13.12	ENSG00000175595
TCF12	transcription factor 12	15q21.3	ENSG00000140262
HSP90AB1	heat shock protein 90kDa alpha (cytosolic), class B member 1	6p21.1	ENSG00000096384
FUBP1	far upstream element (FUSE) binding protein 1	1p31.1	ENSG00000162613
ACSL6	acyl-CoA synthetase long-chain family member 6	5q31.1	ENSG00000164398
USP8	ubiquitin specific peptidase 8	15q21.2	ENSG00000138592
DAXX	death-domain associated protein	6p21.32	ENSG00000204209
RBM15	RNA binding motif protein 15	1p13.3	ENSG00000162775
TBL1XR1	transducin (beta)-like 1 X-linked receptor 1	3q26.32	ENSG00000177565
PCSK7	proprotein convertase subtilisin/kexin type 7	11q23.3	ENSG00000160613
PPFIBP1	PTPRF interacting protein, binding protein 1 (liprin beta 1)	12p11.23, 12p11.22	ENSG00000110841
EZH2	enhancer of zeste 2 polycomb repressive complex 2 subunit	7q36.1	ENSG00000106462

EWSR1	EWS RNA-binding protein 1	22q12.2	ENSG00000182944
CCND2	cyclin D2	12p13.32	ENSG00000118971
MITF	microphthalmia-associated transcription factor	3p13	ENSG00000187098
MAF	v-maf avian musculoaponeurotic fibrosarcoma oncogene homolog	16q23.2	ENSG00000178573
FGFR1OP	FGFR1 oncogene partner	6q27	ENSG00000213066
GAS7	growth arrest-specific 7	17p13.1	ENSG00000007237
TRIM24	tripartite motif containing 24	7q33, 7q34	ENSG00000122779
MYB	v-myb avian myeloblastosis viral oncogene homolog	6q23.3	ENSG00000118513
ECT2L	epithelial cell transforming 2 like	6q24.1	ENSG00000203734
STK11	serine/threonine kinase 11	19p13.3	ENSG00000118046
SEPT6	septin 6	Xq24	ENSG00000125354
NR4A3	nuclear receptor subfamily 4, group A, member 3	9q31.1	ENSG00000119508
TERT	telomerase reverse transcriptase	5p15.33	ENSG00000164362
HNF1A	HNF1 homeobox A	12q24.31	ENSG00000135100
CARS	cysteinyl-tRNA synthetase	11p15.4	ENSG00000110619
SDHA	succinate dehydrogenase complex, subunit A, flavoprotein (Fp)	5p15.33	ENSG00000073578
RAD21	RAD21 homolog (S. pombe)	8q24.11	ENSG00000164754
DDX10	DEAD (Asp-Glu-Ala-Asp) box polypeptide 10	11q22.3	ENSG00000178105
HIF1A	hypoxia inducible factor 1, alpha subunit (basic helix-loop-helix transcription factor)	14q23.2	ENSG00000100644
ETV6	ets variant 6	12p13.2	ENSG00000139083
MSH2	mutS homolog 2	2p21, 2p16.3	ENSG00000095002
ELF4	E74-like factor 4 (ets domain transcription factor)	Xq26.1	ENSG00000102034
EXT1	exostosin glycosyltransferase 1	8q24.11	ENSG00000182197
ARHGAP26	Rho GTPase activating protein 26	5q31.3	ENSG00000145819
BCL6	B-cell CLL/lymphoma 6	3q27.3	ENSG00000113916
RECQL4	RecQ protein-like 4	8q24.3	ENSG00000160957
NF2	neurofibromin 2 (merlin)	22q12.2	ENSG00000186575
HSP90AA1	heat shock protein 90kDa alpha (cytosolic), class A member 1	14q32.31	ENSG00000080824
AKT2	v-akt murine thymoma viral oncogene homolog 2	19q13.2	ENSG00000105221
PTPN11	protein tyrosine phosphatase, non-receptor type 11	12q24.13	ENSG00000179295
PAX7	paired box 7	1p36.13	ENSG00000009709
EPS15	epidermal growth factor receptor pathway substrate 15	1p32.3	ENSG00000085832
MSN	moesin	Xq12	ENSG00000147065
FOXO3	forkhead box O3	6q21	ENSG00000118689
PSIP1	PC4 and SFRS1 interacting protein 1	9p22.3	ENSG00000164985
KIF5B	kinesin family member 5B	10p11.22	ENSG00000170759
HLA-A	major histocompatibility complex, class I, A	6p22.1	ENSG00000206503
SEPT9	septin 9	17q25.3	ENSG00000184640
MLH1	mutL homolog 1	3p22.2	ENSG00000076242
SMAD3	SMAD family member 3	15q22.33	ENSG00000166949
POT1	protection of telomeres 1	7q31.33	ENSG00000128513
NBN	nibrin	8q21.3	ENSG00000104320
ACKR3	atypical chemokine receptor 3	2q37.3	ENSG00000144476
PMS1	PMS1 postmeiotic segregation increased 1 (S. cerevisiae)	2q32.2	ENSG00000064933

TOP 1.00	topoisomerase (DNA) I	20q12	ENSG00000198900
PRF1	perforin 1 (pore forming protein)	10q22.1	ENSG00000180644
LSM14A	LSM14A, SCD6 homolog A (S. cerevisiae)	19q13.11	ENSG00000257103
STAT5B	signal transducer and activator of transcription 5B	17q21.2	ENSG00000173757
LMNA	lamin A/C	1q22	ENSG00000160789
SYK	spleen tyrosine kinase	9q22.2	ENSG00000165025
TNFAIP3	tumor necrosis factor, alpha-induced protein 3	6q23.3	ENSG00000118503
ETV5	ets variant 5	3q27.2	ENSG00000244405
FOXO4	forkhead box O4	Xq13.1	ENSG00000184481
NONO	non-POU domain containing, octamer-binding	Xq13.1	ENSG00000147140
RSPO2	R-spondin 2	8q23.1	ENSG00000147655
IRF4	interferon regulatory factor 4	6p25.3	ENSG00000137265
PPARG	peroxisome proliferator-activated receptor gamma	3p25.2	ENSG00000132170
CDC73	cell division cycle 73	1q31.2	ENSG00000134371
TFRC	transferrin receptor	3q29	ENSG00000072274
LCP1	lymphocyte cytosolic protein 1 (L-plastin)	13q14.13	ENSG00000136167
CEP89	centrosomal protein 89kDa	19q13.11	ENSG00000121289
FOXO1	forkhead box O1	13q14.11	ENSG00000150907
TFE3	transcription factor binding to IGHM enhancer 3	Xp11.23	ENSG00000068323
SMO	smoothened, frizzled class receptor	7q32.1	ENSG00000128602
ZCCHC8	zinc finger, CCHC domain containing 8	12q24.31	ENSG00000033030
CBFA2T3	core-binding factor, runt domain, alpha subunit 2; translocated to, 3	16q24.3	ENSG00000129993
FBXO11	F-box protein 11	2p16.3	ENSG00000138081
IL6ST	interleukin 6 signal transducer	5q11.2	ENSG00000134352
PRRX1	paired related homeobox 1	1q24.2	ENSG00000116132
PAX5	paired box 5	9p13.2	ENSG00000196092
HRAS	Harvey rat sarcoma viral oncogene homolog	11p15.5	ENSG00000174775
CHEK2	checkpoint kinase 2	22q12.1	ENSG00000183765
PHOX2B	paired-like homeobox 2b	4p13	ENSG00000109132
RABEP1	rabaptin, RAB GTPase binding effector protein 1	17p13.2	ENSG00000029725
RPL22	ribosomal protein L22	1p36.31	ENSG00000116251
GPC3	glypican 3	Xq26.2	ENSG00000147257
PATZ1	POZ (BTB) and AT hook containing zinc finger 1	22q12.2	ENSG00000100105
MYCN	v-myc avian myelocytomatosis viral oncogene neuroblastoma derived homolog	2p24.3	ENSG00000134323
TCF3	transcription factor 3	19p13.3	ENSG00000071564
TRIM27	tripartite motif containing 27	6p22.1	ENSG00000204713
BUB1B	BUB1 mitotic checkpoint serine/threonine kinase B	15q15.1	ENSG00000156970
RPL10	ribosomal protein L10	Xq28	ENSG00000147403
EXT2	exostosin glycosyltransferase 2	11p11.2	ENSG00000151348
SMAD2	SMAD family member 2	18q21.1	ENSG00000175387
CREB3L2	cAMP responsive element binding protein 3-like 2	7q33	ENSG00000182158
STAT6	signal transducer and activator of transcription 6, interleukin-4 induced	12q13.3	ENSG00000166888
PHF6	PHD finger protein 6	Xq26.2	ENSG00000156531
AKT1	v-akt murine thymoma viral oncogene homolog 1	14q32.33	ENSG00000142208

FIP1L1	factor interacting with PAPOLA and CPSF1	4q12	ENSG00000145216
MAX	MYC associated factor X	14q23.3	ENSG00000125952
ATIC	5-aminoimidazole-4-carboxamide ribonucleotide formyltransferase/IMP cyclohydrolase	2q35	ENSG00000138363
HNRNPA2B1	heterogeneous nuclear ribonucleoprotein A2/B1	7p15.2	ENSG00000122566
LCK	LCK proto-oncogene, Src family tyrosine kinase	1p35.2	ENSG00000182866
STRN	striatin, calmodulin binding protein	2p22.2	ENSG00000115808
GMPS	guanine monphosphate synthase	3q25.31	ENSG00000163655
DDX5	DEAD (Asp-Glu-Ala-Asp) box helicase 5	17q23.3	ENSG00000108654
HLF	hepatic leukemia factor	17q22	ENSG00000108924
FAM131B	family with sequence similarity 131, member B	7q34	ENSG00000159784
FUS	FUS RNA binding protein	16p11.2	ENSG00000089280
SFPQ	splicing factor proline/glutamine-rich	1p34.3	ENSG00000116560
CRTC1	CREB regulated transcription coactivator 1	19p13.11	ENSG00000105662
HMGA2	high mobility group AT-hook 2	12q14.3	ENSG00000149948
RHOA	ras homolog family member A	3p21.31	ENSG00000067560
PBX1	pre-B-cell leukemia homeobox 1	1q23.3	ENSG00000185630
EIF3E	eukaryotic translation initiation factor 3, subunit E	8q23.1	ENSG00000104408
ARNT	aryl hydrocarbon receptor nuclear translocator	1q21.3	ENSG00000143437
SOX2	SRY (sex determining region Y)-box 2	3q26.33	ENSG00000181449
HOOK3	hook microtubule-tethering protein 3	8p11.21	ENSG00000168172
KCNJ5	potassium channel, inwardly rectifying subfamily J, member 5	11q24.3	ENSG00000120457
RAC1	ras-related C3 botulinum toxin substrate 1 (rho family, small GTP binding protein Rac1)	7p22.1	ENSG00000136238
LEF1	lymphoid enhancer-binding factor 1	4q25	ENSG00000138795
RAF1	Raf-1 proto-oncogene, serine/threonine kinase	3p25.2	ENSG00000132155
PICALM	phosphatidylinositol binding clathrin assembly protein	11q14.2	ENSG00000073921
GOLGA5	golgin A5	14q32.12	ENSG00000066455
FLCN	folliculin	17p11.2	ENSG00000154803
DDX6	DEAD (Asp-Glu-Ala-Asp) box helicase 6	11q23.3	ENSG00000110367
MYC	v-myc avian myelocytomatosis viral oncogene homolog	8q24.21	ENSG00000136997
MALT1	MALT1 paracaspase	18q21.32	ENSG00000172175
CXCR4	chemokine (C-X-C motif) receptor 4	2q22.1	ENSG00000121966
CCDC6	coiled-coil domain containing 6	10q21.2	ENSG00000108091
ETNK1	ethanolamine kinase 1	12p12.1	ENSG00000139163
NFKB2	nuclear factor of kappa light polypeptide gene enhancer in B-cells 2 (p49/p100)	10q24.32	ENSG00000077150
SLC45A3	solute carrier family 45, member 3	1q32.1	ENSG00000158715
TFEB	transcription factor EB	6p21.1	ENSG00000112561
GNAQ	guanine nucleotide binding protein (G protein), q polypeptide	9q21.2	ENSG00000156052
ELL	elongation factor RNA polymerase II	19p13.11	ENSG00000105656
ZNF384	zinc finger protein 384	12p13.31	ENSG00000126746
RAP1GDS1	RAP1, GTP-GDP dissociation stimulator 1	4q23	ENSG00000138698
XPC	xeroderma pigmentosum, complementation group C	3p25.1	ENSG00000154767
PRKACA	protein kinase, cAMP-dependent, catalytic, alpha	19p13.12	ENSG00000072062
RPL5	ribosomal protein L5	1p22.1	ENSG00000122406

MEN1	multiple endocrine neoplasia I	11q13.1	ENSG00000133895
FNBP1	formin binding protein 1	9q34.11	ENSG00000187239
TRAF7	TNF receptor-associated factor 7, E3 ubiquitin protein ligase	16p13.3	ENSG00000131653
PAX8	paired box 8	2q14.1	ENSG00000125618
MLLT1	myeloid/lymphoid or mixed-lineage leukemia (trithorax homolog, Drosophila); translocated to, 1	19p13.3	ENSG00000130382
BMPR1A	bone morphogenetic protein receptor, type IA	10q23.2	ENSG00000107779
MAP2K1	mitogen-activated protein kinase kinase 1	15q22.31	ENSG00000169032
EZR	ezrin	6q25.3	ENSG00000092820
TMPRSS2	transmembrane protease, serine 2	21q22.3	ENSG00000184012
WAS	Wiskott-Aldrich syndrome	Xp11.23	ENSG00000015285
RARA	retinoic acid receptor, alpha	17q21.2	ENSG00000131759
BCL7A	B-cell CLL/lymphoma 7A	12q24.31	ENSG00000110987
PRKAR1A	protein kinase, cAMP-dependent, regulatory, type I, alpha	17q24.2	ENSG00000108946
TPM3	tropomyosin 3	1q21.3	ENSG00000143549
MPL	MPL proto-oncogene, thrombopoietin receptor	1p34.2	ENSG00000117400
CCNE1	cyclin E1	19q12	ENSG00000105173
SMARCB1	SWI/SNF related, matrix associated, actin dependent regulator of chromatin, subfamily b, member 1	22q11.23	ENSG00000099956
GNA11	guanine nucleotide binding protein (G protein), alpha 11 (Gq class)	19p13.3	ENSG00000088256
FANCC	Fanconi anemia, complementation group C	9q22.32	ENSG00000158169
SUZ12	SUZ12 polycomb repressive complex 2 subunit	17q11.2	ENSG00000178691
CHN1	chimerin 1	2q31.1	ENSG00000128656
GATA2	GATA binding protein 2	3q21.3	ENSG00000179348
SUFU	suppressor of fused homolog (Drosophila)	10q24.32	ENSG00000107882
CCND1	cyclin D1	11q13.3	ENSG00000110092
KLK2	kallikrein-related peptidase 2	19q13.33	ENSG00000167751
ACVR1	activin A receptor, type I	2q24.1	ENSG00000115170
GOPC	golgi-associated PDZ and coiled-coil motif containing	6q22.1	ENSG00000047932
MYCL	v-myc avian myelocytomatosis viral oncogene lung carcinoma derived homolog	1p34.2	ENSG00000116990
JUN	jun proto-oncogene	1p32.1	ENSG00000177606
PPP6C	protein phosphatase 6, catalytic subunit	9q33.3	ENSG00000119414
NKX2-1	NK2 homeobox 1	14q13.3	ENSG00000136352
TNFRSF14	tumor necrosis factor receptor superfamily, member 14	1p36.32	ENSG00000157873
ASPSCR1	alveolar soft part sarcoma chromosome region, candidate 1	17q25.3	ENSG00000169696
WIF1	WNT inhibitory factor 1	12q14.3	ENSG00000156076
SMARCE1	SWI/SNF related, matrix associated, actin dependent regulator of chromatin, subfamily e, member 1	17q21.2	ENSG00000073584
MUTYH	mutY homolog	1p34.1	ENSG00000132781
TAL1	T-cell acute lymphocytic leukemia 1	1p33	ENSG00000162367
HOXA9	homeobox A9	7p15.2	ENSG00000078399
ACSL3	acyl-CoA synthetase long-chain family member 3	2q36.1	ENSG00000123983
REL	v-rel avian reticuloendotheliosis viral oncogene homolog	2p16.1	ENSG00000162924
NAB2	NGFI-A binding protein 2 (EGR1 binding protein 2)	12q13.3	ENSG00000166886
NDRG1	N-myc downstream regulated 1	8q24.22	ENSG00000104419
CDKN1B	cyclin-dependent kinase inhibitor 1B (p27, Kip1)	12p13.1	ENSG00000111276

BIRC3	baculoviral IAP repeat containing 3	11q22.2	ENSG00000023445
RSPO3	R-spondin 3	6q22.33	ENSG00000146374
MYOD1	myogenic differentiation 1	11p15.1	ENSG00000129152
TPM4	tropomyosin 4	19p13.12, 19p13.11	ENSG00000167460
SS18L1	synovial sarcoma translocation gene on chromosome 18-like 1	20q13.33	ENSG00000184402
ALDH2	aldehyde dehydrogenase 2 family (mitochondrial)	12q24.12	ENSG00000111275
CRTC3	CREB regulated transcription coactivator 3	15q26.1	ENSG00000140577
FH	fumarate hydratase	1q43	ENSG00000091483
MSI2	musashi RNA-binding protein 2	17q22	ENSG00000153944
PIM1	Pim-1 proto-oncogene, serine/threonine kinase	6p21.2	ENSG00000137193
DEK	DEK proto-oncogene	6p22.3	ENSG00000124795
KLF4	Kruppel-like factor 4 (gut)	9q31.2	ENSG00000136826
KLF6	Kruppel-like factor 6	10p15.2	ENSG00000067082
DDB2	damage-specific DNA binding protein 2, 48kDa	11p11.2	ENSG00000134574
NCKIPSD	NCK interacting protein with SH3 domain	3p21.31	ENSG00000213672
IDH2	isocitrate dehydrogenase 2 (NADP+), mitochondrial	15q26.1	ENSG00000182054
H3F3B	H3 histone, family 3B (H3.3B)	17q25.1	ENSG00000132475
PRCC	papillary renal cell carcinoma (translocation-associated)	1q23.1	ENSG00000143294
SEPT5	septin 5	22q11.21	ENSG00000184702
CDK6	cyclin-dependent kinase 6	7q21.2	ENSG00000105810
SS18	synovial sarcoma translocation, chromosome 18	18q11.2	ENSG00000141380
RPN1	ribophorin I	3q21.3	ENSG00000163902
CREB3L1	cAMP responsive element binding protein 3-like 1	11p11.2	ENSG00000157613
GATA1	GATA binding protein 1 (globin transcription factor 1)	Xp11.23	ENSG00000102145
CBFB	core-binding factor, beta subunit	16q22.1	ENSG00000067955
CANT1	calcium activated nucleotidase 1	17q25.3	ENSG00000171302
SMARCD1	SWI/SNF related, matrix associated, actin dependent regulator of chromatin, subfamily d, member 1	12q13.12	ENSG00000066117
PWWP2A	PWWP domain containing 2A	5q33.3	ENSG00000170234
BCL3	B-cell CLL/lymphoma 3	19q13.32	ENSG00000069399
NPM1	nucleophosmin (nucleolar phosphoprotein B23, numatrin)	5q35.1	ENSG00000181163
MDM2	MDM2 proto-oncogene, E3 ubiquitin protein ligase	12q15	ENSG00000135679
BCL2	B-cell CLL/lymphoma 2	18q21.33	ENSG00000171791
ETV4	ets variant 4	17q21.31	ENSG00000175832
FAS	Fas cell surface death receptor	10q23.31	ENSG00000026103
HOXD13	homeobox D13	2q31.1	ENSG00000128714
FANCG	Fanconi anemia, complementation group G	9p13.3	ENSG00000221829
FCGR2B	Fc fragment of IgG, low affinity IIb, receptor (CD32)	1q23.3	ENSG00000072694
PTK6	protein tyrosine kinase 6	20q13.33	ENSG00000101213
MAP2K2	mitogen-activated protein kinase kinase 2	19p13.3	ENSG00000126934
HOXC13	homeobox C13	12q13.13	ENSG00000123364
MLF 1.00	myeloid leukemia factor 1	3q25.32	ENSG00000178053
JAZF1	JAZF zinc finger 1	7p15.2, 7p15.1	ENSG00000153814
HERPUD1	homocysteine-inducible, endoplasmic reticulum stress-inducible, ubiquitin-like domain member 1	16q13	ENSG00000051108

PDGFB	platelet-derived growth factor beta polypeptide	22q13.1	ENSG00000100311
MAFB	v-maf avian musculoaponeurotic fibrosarcoma oncogene homolog B	20q12	ENSG00000204103
HOXA13	homeobox A13	7p15.2	ENSG00000106031
RHOH	ras homolog family member H	4p14	ENSG00000168421
ABI1	abl-interactor 1	10p12.1	ENSG00000136754
CALR	calreticulin	19p13.13	ENSG00000179218
RAD51B	RAD51 paralog B	14q24.1	ENSG00000182185
HOXA11	homeobox A11	7p15.2	ENSG00000005073
SET	SET nuclear proto-oncogene	9q34.11	ENSG00000119335
CLP 1	cleavage and polyadenylation factor I subunit 1	11q12.1	ENSG00000172409
HEY1	hes-related family bHLH transcription factor with YRPW motif 1	8q21.13	ENSG00000164683
CDX2	caudal type homeobox 2	13q12.2	ENSG00000165556
SRSF2	serine/arginine-rich splicing factor 2	17q25.1	ENSG00000161547
SH3GL1	SH3-domain GRB2-like 1	19p13.3	ENSG00000141985
HOXC11	homeobox C11	12q13.13	ENSG00000123388
NT5C2	5'-nucleotidase, cytosolic II	10q24.32, 10q24.33	ENSG00000076685
SH2B3	SH2B adaptor protein 3	12q24.12	ENSG00000111252
CCNB1IP1	cyclin B1 interacting protein 1, E3 ubiquitin protein ligase	14q11.2	ENSG00000100814
ELK4	ELK4, ETS-domain protein (SRF accessory protein 1)	1q32.1	ENSG00000158711
MYD88	myeloid differentiation primary response 88	3p22.2	ENSG00000172936
LASP1	LIM and SH3 protein 1	17q12	ENSG00000002834
MUC1	mucin 1, cell surface associated	1q22	ENSG00000185499
OLIG2	oligodendrocyte lineage transcription factor 2	21q22.11	ENSG00000205927
VTI1A	vesicle transport through interaction with t-SNAREs 1A	10q25.2	ENSG00000151532
OMD	osteomodulin	9q22.31	ENSG00000127083
POU2AF1	POU class 2 associating factor 1	11q23.1	ENSG00000110777
FOXL2	forkhead box L2	3q22.3	ENSG00000183770
SSX1	synovial sarcoma, X breakpoint 1	Xp11.23	ENSG00000126752
CCND3	cyclin D3	6p21.1	ENSG00000112576
YWHAE	tyrosine 3-monooxygenase/tryptophan 5-monooxygenase activation protein, epsilon	17p13.3	ENSG00000108953
SDHC	succinate dehydrogenase complex, subunit C, integral membrane protein, 15kDa	1q23.3	ENSG00000143252
MDM4	MDM4, p53 regulator	1q32.1	ENSG00000198625
CBLC	Cbl proto-oncogene C, E3 ubiquitin protein ligase	19q13.32	ENSG00000142273
TCEA1	transcription elongation factor A (SII), 1	8q11.23	ENSG00000187735
TTL	tubulin tyrosine ligase	2q14.1	ENSG00000114999
NUTM2A	NUT family member 2A	10q23.2	ENSG00000184923
TFG	TRK-fused gene	3q12.2	ENSG00000114354
FANCE	Fanconi anemia, complementation group E	6p21.31	ENSG00000112039
SBDS	Shwachman-Bodian-Diamond syndrome	7q11.21	ENSG00000126524
WWTR1	WW domain containing transcription regulator 1	3q25.1	ENSG00000018408
CD274	CD274 molecule	9p24.1	ENSG00000120217
ATF1	activating transcription factor 1	12q13.12	ENSG00000123268
POU5F1	POU class 5 homeobox 1	6p21.33	ENSG00000204531

SRSF3	serine/arginine-rich splicing factor 3	6p21.31, 6p21.2	ENSG00000112081
TFPT	TCF3 (E2A) fusion partner (in childhood Leukemia)	19q13.42	ENSG00000105619
DNAJB1	DnaJ (Hsp40) homolog, subfamily B, member 1	19p13.12	ENSG00000132002
MNX1	motor neuron and pancreas homeobox 1	7q36.3	ENSG00000130675
TLX1	T-cell leukemia homeobox 1	10q24.31	ENSG00000107807
LMO2	LIM domain only 2 (rhombotin-like 1)	11p13	ENSG00000135363
BTG1	B-cell translocation gene 1, anti-proliferative	12q21.33	ENSG00000133639
CD79A	CD79a molecule, immunoglobulin-associated alpha	19q13.2	ENSG00000105369
CHIC2	cysteine-rich hydrophobic domain 2	4q12	ENSG00000109220
CD74	CD74 molecule, major histocompatibility complex, class II invariant chain	5q33.1	ENSG00000019582
TNFRSF17	tumor necrosis factor receptor superfamily, member 17	16p13.13	ENSG00000048462
SDC4	syndecan 4	20q13.12	ENSG00000124145
MLLT11	myeloid/lymphoid or mixed-lineage leukemia (trithorax homolog, Drosophila); translocated to, 11	1q21.3	ENSG00000213190
PDCD1LG2	programmed cell death 1 ligand 2	9p24.1	ENSG00000197646
XPA	xeroderma pigmentosum, complementation group A	9q22.33	ENSG00000136936
FANCF	Fanconi anemia, complementation group F	11p14.3	ENSG00000183161
TLX3	T-cell leukemia homeobox 3	5q35.1	ENSG00000164438
CDK4	cyclin-dependent kinase 4	12q14.1	ENSG00000135446
CD79B	CD79b molecule, immunoglobulin-associated beta	17q23.3	ENSG00000007312
CREB1	cAMP responsive element binding protein 1	2q33.3	ENSG00000118260
KDSR	3-ketodihydrosphingosine reductase	18q21.33	ENSG00000119537
NFKBIE	nuclear factor of kappa light polypeptide gene enhancer in B-cells inhibitor, epsilon	6p21.1	ENSG00000146232
H3F3A	H3 histone, family 3A	1q42.12	ENSG00000163041
CDKN2C	cyclin-dependent kinase inhibitor 2C (p18, inhibits CDK4)	1p32.3	ENSG00000123080
FHIT	fragile histidine triad	3p14.2	ENSG00000189283
ZRSR2	zinc finger (CCCH type), RNA-binding motif and serine/arginine rich 2	Xp22.2	ENSG00000169249
TCL1A	T-cell leukemia/lymphoma 1A	14q32.13	ENSG00000100721
BCL10	B-cell CLL/lymphoma 10	1p22.3	ENSG00000142867
SDHB	succinate dehydrogenase complex, subunit B, iron sulfur (Ip)	1p36.13	ENSG00000117118
LMO1	LIM domain only 1 (rhombotin 1)	11p15.4	ENSG00000166407
CNBP	CCHC-type zinc finger, nucleic acid binding protein	3q21.3	ENSG00000169714
HOXD11	homeobox D11	2q31.1	ENSG00000128713
SDHAF2	succinate dehydrogenase complex assembly factor 2	11q12.2	ENSG00000167985
DDIT3	DNA-damage-inducible transcript 3	12q13.3	ENSG00000175197
MDS2	myelodysplastic syndrome 2 translocation associated	1p36.12, 1p36.11	ENSG00000197880
IL2	interleukin 2	4q27	ENSG00000109471
SDHD	succinate dehydrogenase complex, subunit D, integral membrane protein	11q23.1	ENSG00000204370
PAFAH1B2	platelet-activating factor acetylhydrolase 1b, catalytic subunit 2 (30kDa)	11q23.3	ENSG00000168092
HMGA1	high mobility group AT-hook 1	6p21.31	ENSG00000137309
MTCP1	mature T-cell proliferation 1	Xq28	ENSG00000214827
CEBPA	CCAAT/enhancer binding protein (C/EBP), alpha	19q13.11	ENSG00000245848

TAL2	T-cell acute lymphocytic leukemia 2	9q31.2	ENSG00000186051
SOCS1	suppressor of cytokine signaling 1	16p13.13	ENSG00000185338
FSTL3	follistatin-like 3 (secreted glycoprotein)	19p13.3	ENSG00000070404
LYL1	lymphoblastic leukemia associated hematopoiesis regulator 1	19p13.13	ENSG00000104903
CHCHD7	coiled-coil-helix-coiled-coil-helix domain containing 7	8q12.1	ENSG00000170791
RMI2	RecQ mediated genome instability 2	16p13.13	ENSG00000175643
FEV	FEV (ETS oncogene family)	2q35	ENSG00000163497
COX6C	cytochrome c oxidase subunit VIc	8q22.2	ENSG00000164919
C15orf65	chromosome 15 open reading frame 65	15q21.3	ENSG00000261652

Table S5: Results of SVM regression with various kernels

Table S5 Results of SVM regression with various kernels

kernel	Pearson's correlation	Spearman's correlation	RMSE
RBF	0.313±0.069	0.213±0.070	3.105±0.052
polynomial	0.076±0.019	**0.273±0.037**	**3.038±0.060**
sigmoid	**0.336±0.078**	0.230±0.071	3.115±0.053

Table S6: Results of random forest with various number of trees

Table S6 Results of random forest with various number of trees

Num. of trees	Pearson's correlation	Spearman's correlation	RMSE
50	0.863±0.002	0.838±0.003	1.445±0.008
100	**0.864±0.001**	**0.839±0.003**	**1.441±0.008**
200	0.864±0.001	0.839±0.003	1.441±0.008

Table S7: Results of Lasso regression with various alpha

Table S7 Results of Lasso regression with various alpha

alpha	Pearson's correlation	Spearman's correlation	RMSE
0.01	**0.893±0.002**	**0.873±0.002**	**1.284±0.007**
0.1	0.818±0.002	0.777±0.003	1.715±0.009
0.5	0.558±0.002	0.496±0.007	2.556±0.014
